# Pressure‐Tuning Photothermal Synergy to Optimize the Photoelectronic Properties in Amorphous Halide Perovskite Cs_3_Bi_2_I_9_

**DOI:** 10.1002/advs.202205837

**Published:** 2022-12-29

**Authors:** Zonglun Li, Binxia Jia, Sixue Fang, Quanjun Li, Fuyu Tian, Haiyan Li, Ran Liu, Yucheng Liu, Lijun Zhang, Shengzhong (Frank) Liu, Bingbing Liu

**Affiliations:** ^1^ State Key Laboratory of Superhard Materials Jilin University Changchun 130012 P. R. China; ^2^ Key Laboratory of Applied Surface and Colloid Chemistry National Ministry of Education Shaanxi Engineering Lab for Advanced Energy Technology School of Materials Science and Engineering Shaanxi Normal University Xi'an 710119 P. R. China; ^3^ Key Laboratory of Automobile Materials of MOE and School of Materials Science and Engineering Jilin University Changchun 130012 P. R. China

**Keywords:** amorphous materials, broadband photoresponse, Cs_3_Bi_2_I_9_, halide perovskites, high pressure

## Abstract

Effective modification of the structure and properties of halide perovskites via the pressure engineering strategy has attracted enormous interest in the past decade. However, sufficient effort and insights regarding the potential properties and applications of the high‐pressure amorphous phase are still lacking. Here, the superior and tunable photoelectric properties that occur in the pressure‐induced amorphization process of the halide perovskite Cs_3_Bi_2_I_9_ are demonstrated. With increasing pressure, the photocurrent with xenon lamp illumination exhibits a rapid increase and achieves an almost five orders of magnitude increment compared to its initial value. Impressively, a broadband photoresponse from 520 to 1650 nm with an optimal responsivity of 6.81 mA W^−1^ and fast response times of 95/96 ms at 1650 nm is achieved upon successive compression. The high‐gain, fast, broadband, and dramatically enhanced photoresponse properties of Cs_3_Bi_2_I_9_ are the result of comprehensive photoconductive and photothermoelectric mechanisms, which are associated with enhanced orbital coupling caused by an increase in Bi—I interactions in the [BiI_6_]^3−^ cluster, even in the amorphous state. These findings provide new insights for further exploring the potential properties and applications of amorphous perovskites.

## Graphical abstract

The tunable and broadband photoresponse from 520 to 1650 nm with an optimal responsivity of 6.81 mA W^−1^ and fast response times of 95/96 ms at 1650 nm is achieved in the pressure‐induced amorphization process of the halide perovskite Cs_3_Bi_2_I_9_, which provides some new insights into further investigating the unexplored properties and applications of amorphous perovskites.

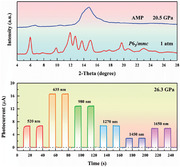

## 1 Introduction

Metal halide perovskites are currently the subject of extensive investigations owing to their distinguished properties and attractive prospects for various potential applications, including high‐efficiency solar cells,^[^ ^1^, ^2^ ^]^ radiation detection,^[^ ^3^, ^4^ ^]^ light‐emitting diodes (LEDs),^[^ ^5^, ^6^ ^]^ lasing,^[^ ^7^ ^]^ and photodetectors,^[^ ^8^, ^9^, ^10^ ^]^ especially diverse applications of photodetector in daily life and military field.^[^ ^11^, ^12^ ^]^ Albeit the low stability, poor flexibility, and toxic heavy metals (Pb, Hg, Cd, etc.) are fundamental challenges for further practical applications. Tremendous progress has been achieved in exploring and optimizing metal halide perovskites during the past decade. Among the various perovskites, bismuth‐based halide perovskites with high stability, low toxicity and the isoelectronic nature of Bi^+^ compared to Pb^+^ have attracted tremendous interest as photoelectric materials.^[^ ^13^, ^14^, ^15^, ^16^ ^]^ In particular, Cs_3_Bi_2_I_9_ has already been extensively employed for the fabrication of high‐performance photodetectors with excellent X‐ray imaging capability,^[^ ^14^ ^]^ impressive on/off ratio,^[^ ^15^ ^]^ and broadband photoresponse (254–1064 nm).^[^ ^16^ ^]^ Regrettably, except when used to assemble complicated heterojunctions, the intrinsic photoresponse of Cs_3_Bi_2_I_9_ is limited to within the UV‒vis waveband owing to its wide indirect bandgap of 1.9–2.8 eV.^[^ ^8^, ^16^ ^]^ Consequently, further exploring new strategies to extend the photoresponse range to enrich the functionalities and applications of Cs_3_Bi_2_I_9_ is imperative.

High pressure is a straightforward and significant approach to regulate the crystal and electronic structure of materials, and great accomplishments have been achieved in optimizing the properties of halide perovskite materials,^[^ ^17^, ^18^, ^19^, ^20^, ^21^, ^22^, ^23^, ^24^, ^25^ ^]^ such as enhanced structural stability,^[^ ^17^ ^]^ pressure‐induced and enhanced emission,^[^ ^18^ ^]^ piezochromism,^[^ ^19^ ^]^ captured high‐pressure phase down to ambient conditions,^[^ ^21^ ^]^ and regulated photocurrents.^[^ ^17^, ^23^, ^24^, ^25^ ^]^ In particular, a remarkably enhanced photocurrent by over three orders of magnitude via a pressure‐reduced exciton binding energy in the all‐inorganic perovskite Cs_2_PbI_2_Cl_2_^[^ ^23^ ^]^ and even an extended spectral response range from the visible to near‐infrared waveband were achieved in bulk iodine, PbI_2_ and hypervalent CsI_3_.^[^ ^26^, ^27^, ^28^ ^]^ These efforts have indicated that pressure application is an efficient strategy to optimize photoelectric properties such as the photocurrent and detection bandwidth dominated by the photoconductive mechanism. Intriguingly, many halide perovskites, such as Cs_3_Bi_2_I_9_, exhibit successive bandgap reduction with increasing pressure,^[^ ^29^, ^30^, ^31^ ^]^ which hints at the possibility of extending the spectral response range to a longer waveband.

Notably, some halide perovskites generally undergo pressure‐induced amorphization accompanied by changes in optical and electrical properties.^[^ ^18^, ^19^, ^20^, ^22^, ^23^, ^24^, ^25^ ^]^ The thermal behavior of disordered amorphous materials is quite different from that of ordered crystalline materials,^[^ ^32^, ^33^, ^34^ ^]^ generally possessing lower thermal conductivity and superior flexibility, thus contributing to a considerable thermoelectric energy conversion and practical applications in flexible devices.^[^ ^34^, ^35^, ^36^ ^]^ The thermoelectric applications require excellent electrical conductivity, which is a considerable challenge in amorphous materials,^[^ ^32^ ^]^ and generally, the wide bandgap results in photoelectric applications mainly being limited to the solar‐blind region.^[^ ^37^, ^38^ ^]^ Fortunately, this can be addressed in amorphous perovskites owing to the bandgap reduction and metallization occurring in the pressurization process.^[^ ^22^, ^29^ ^]^ As a critical photoresponse mechanism, the photothermoelectric effect combines photothermal conversion processes and the thermoelectric effect and can achieve a broadband photoresponse with a self‐powered operation capability under zero external bias.^[^ ^39^, ^40^, ^41^ ^]^ The photothermal conversion is determined by the optical absorption and heat capacity.^[^ ^39^ ^]^ Not only highly pressure‐tunable absorption coefficients but also a lower heat capacity can be achieved in amorphous perovskites,^[^ ^22^, ^32^, ^34^ ^]^ which are responsible for the excellent photothermal conversion efficiency in amorphous perovskites. Therefore, exceptional photoelectric properties dominated by the photothermoelectric mechanism can be expected. Nevertheless, amorphous perovskites are usually overlooked, and further investigation is urgently required to obtain more valuable insights into the various potential properties and applications, especially for photodetection.

In this work, we demonstrate the fascinating photoelectric properties of the amorphous halide perovskite Cs_3_Bi_2_I_9_ under high pressure. Significant enhancement in the photocurrent with xenon lamp illumination by almost five orders of magnitude is achieved upon compression, accompanying the amorphization process. Simultaneously, the spectral response range is successfully extended from the visible to optical communication waveband (1650 nm) with successively increasing pressure. Upon further compression, the fast, stable, high‐gain, and rapidly increasing photoresponse is the result of synergy of the photoconductive and photothermoelectric effects. The superb and tunable photoelectric properties of amorphous Cs_3_Bi_2_I_9_ are anticipated to inspire extensive interest in the study of amorphous perovskites.

## 2 Results and Discussion

**Figure 1:**
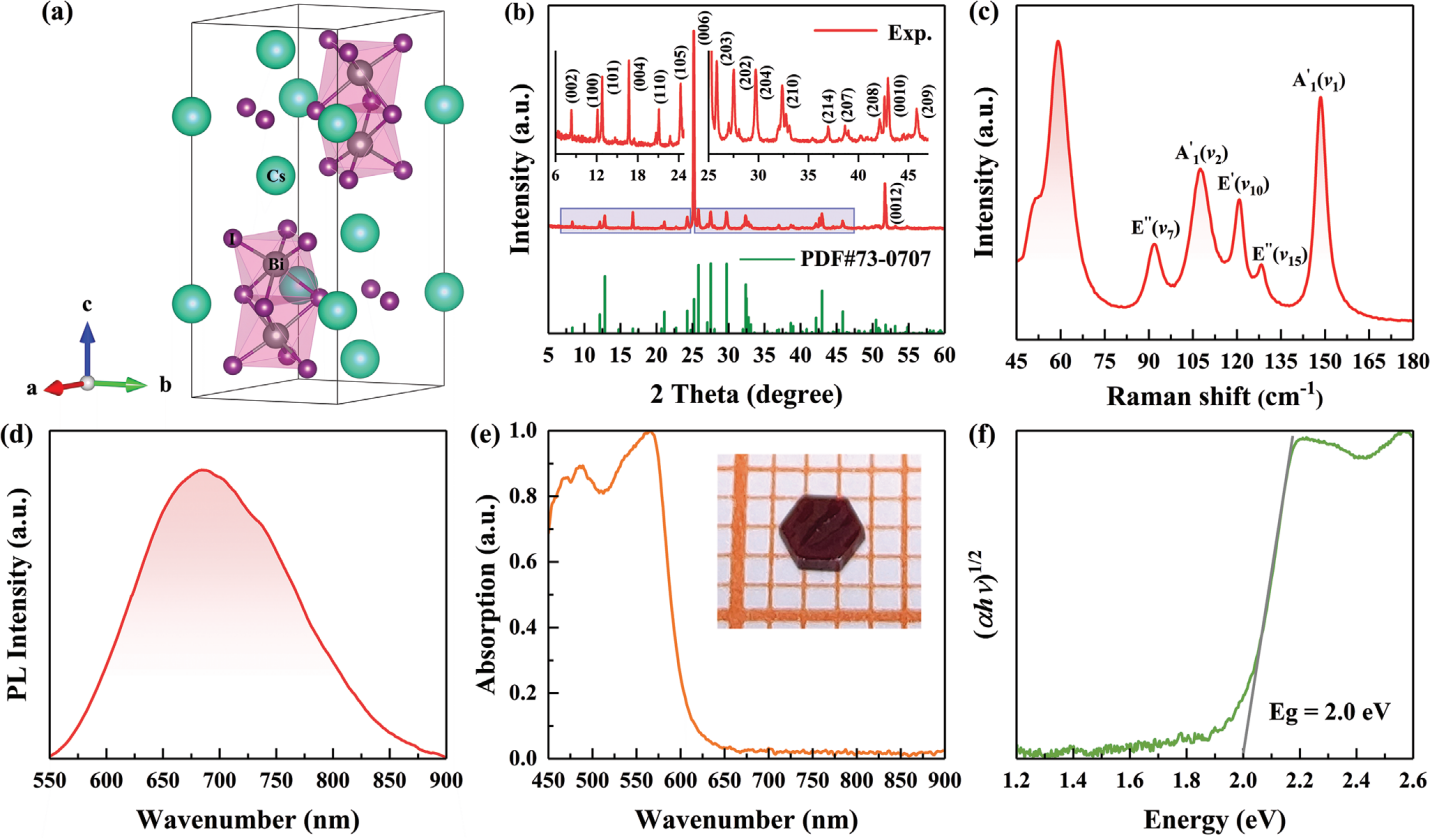
Sample characterization of Cs_3_Bi_2_I_9_ at ambient conditions. a) Crystal structure of *P*6_3_ */mmc* phase Cs_3_Bi_2_I_9_. b) XRD pattern of Cs_3_Bi_2_I_9_. c) Raman spectrum of Cs_3_Bi_2_I_9_. d) PL spectrum of Cs_3_Bi_2_I_9_. e) Optical absorption spectrum of Cs_3_Bi_2_I_9_. The inset shows the optical photograph of Cs_3_Bi_2_I_9_ single crystal sample. f) Tauc plot for the optical absorption spectrum of Cs_3_Bi_2_I_9_ to determine the bandgap.

Cs_3_Bi_2_I_9_ adopts a hexagonal structure with the space group *P*6_3_/*mmc* under ambient conditions.^[^ ^13^, ^29^ ^]^ As illustrated in **Figure** **1**a, the crystal structure of Cs_3_Bi_2_I_9_ consists of a [Bi_2_I_9_]^3−^ dioctahedral cluster and Cs^+^ cations. Two neighboring [BiI_6_]^3−^ octahedra share a face along the *c*‐axis direction to form a [Bi_2_I_9_]^3−^ dioctahedral cluster, and the hexagonal channels are filled by Cs^+^ cations to form a 0D molecular salt structure. There are two groups of Bi—I bonds in the [Bi_2_I_9_]^3−^ dioctahedral cluster: the three long bridging Bi—I bonds involve the I atoms that share bonds with two Bi atoms, and the six rigid terminal Bi—I bonds are oriented away from the shared face.^[^ ^13^, ^14^ ^]^ Lattice constants of *a* = *b* = 8.41 Å and *c* = 21.18 Å were determined according to the powder X‐ray diffraction (XRD) pattern data (Figure 1b), which are consistent with the standard PDF card No. 73‐0707 and previous literature reports.^[^ ^14^ ^]^ The in situ high‐pressure XRD results reveal that the hexagonal phase of Cs_3_Bi_2_I_9_ begins to transform into an amorphous structure at ≈12.1 GPa, as evidenced by the broadening and disappearance of XRD peaks (Figure S1a, Supporting Information). The amorphization originates from disordered rearrangement of the [Bi_2_I_9_]^3−^ dioctahedral cluster. The hexagonal and amorphous phases coexist up to 21 GPa. When the pressure exceeds 21 GPa, all diffraction peaks entirely disappear, leaving merely a broad band, which indicates that the hexagonal phase Cs_3_Bi_2_I_9_ is completely amorphized (Figure S1a, Supporting Information).^[^ ^29^ ^]^

The Raman spectrum of Cs_3_Bi_2_I_9_ under ambient conditions is illustrated in Figure 1c. Seven distinct peaks are observed at 51.6, 59.3, 91.9, 107.7, 120.8, 128.3, and 148.5 cm^−1^. The dominant vibrations of the molecular salt structure of Cs_3_Bi_2_I_9_ originate from the intensively bound [Bi_2_I_9_]^3−^ cluster. Within the [Bi_2_I_9_]^3−^ cluster, the two vibrational modes are the terminal Bi—I stretching mode and bridging Bi—I stretching mode. The Raman peaks in the ranges of 120.8–148.5 cm^−1^ and 91.9–107.7 cm^−1^ are attributable to the terminal Bi—I stretching modes and bridging Bi—I stretching modes, respectively. The terminal Bi—I symmetric stretching mode is A″_1_(*v*_1_) at 148.5 cm^−1^, and its antisymmetric partners are E″″ (*v*_15_) at 128.3 cm^−1^ and E″(*v*_10_) at 120.8 cm^−1^. The bridging Bi—I symmetric stretching mode A_1_^1^(*v*_2_) and its antisymmetric mode E″″ (*v*_7_) are at 107.7 cm^−1^ and 91.9 cm^−1^, respectively. The lower energy modes at 51.6 and 59.3 cm^−1^ are associated with the various bending modes and ionic interactions of the [Bi_2_I_9_]^3−^ cluster and nearby Cs^+^ cations.^[^ ^13^ ^]^

The Cs_3_Bi_2_I_9_ sample was further characterized by photoluminescence (PL) spectroscopy and optical absorption spectroscopy. As shown in Figure 1d, the narrow PL peak indicates the low defect density of the sample.^[^ ^15^ ^]^ The optical absorption intensity rapidly attenuated along the absorption edge is shown in Figure 1e. Cs_3_Bi_2_I_9_ is an indirect bandgap semiconductor, and its optical bandgap can be extrapolated from the absorption spectrum via the Tauc equation (*hνα*)^1/2^ = *A*(*hv−E*_g_), where *h* represents Planck's constant, *v* is the photon frequency, *α* is the absorption coefficient, *E*_g_ is the bandgap, and *A* is a constant. As illustrated in Figure 1f, the bandgap of the Cs_3_Bi_2_I_9_ sample can be estimated as 2.0 eV, agreeing with prior reports.^[^ ^13^, ^14^, ^29^ ^]^

**Figure 2:**
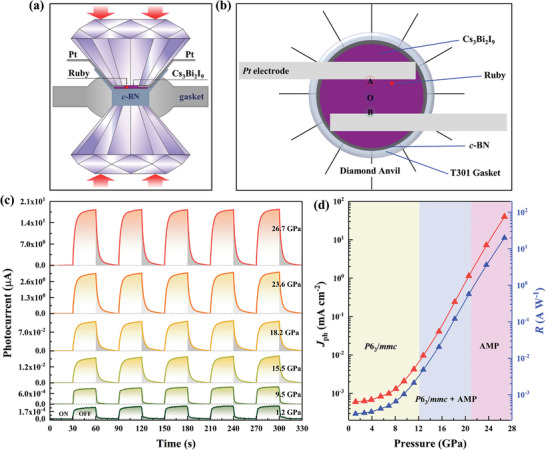
Photoelectric properties of Cs_3_Bi_2_I_9_ with xenon lamp illumination under high pressure. a) DAC setup with components annotated for in situ photoelectric measurements under high pressure. b) Schematic of the sample chamber configuration for the photoelectric measurements in the DAC. c) Photocurrent curves of Cs_3_Bi_2_I_9_ under xenon lamp illumination at selected pressures; the light spot size is considerably larger than the active device area. d) Variations in the photocurrent density *J*_ph_ and responsivity *R* of Cs_3_Bi_2_I_9_ as a function of pressure under a 10 V bias. AMP is an abbreviation for amorphous phase.

As schematically illustrated in **Figure** **2**a,b, to systematically investigate the variations in the photoelectric properties of Cs_3_Bi_2_I_9_ upon compression, we fabricated an in situ high‐pressure photoelectric measurement setup via a two‐point probe method within a diamond anvil cell (DAC). The labeled O position corresponds to the center of the device, and the labeled A and B positions are located near the two interfaces between the sample and the two probes. The photoelectric properties of Cs_3_Bi_2_I_9_ under global xenon lamp illumination were evaluated and are displayed in Figure 2c and Figure S2, Supporting Information. Figure S2a, Supporting Information, depicts the symmetric and linear *I–V* curves of the Cs_3_Bi_2_I_9_ device under illumination and in the dark at the initial pressure, which shows the significant enhancement in the photocurrent with increasing external bias (Figure S2b, Supporting Information), which evidences good ohmic contact between the Cs_3_Bi_2_I_9_ sample and Pt electrodes. This feature persists upon further compression (Figure S2c,d, Supporting Information).

The responsivity (*R*) is a figure of merit for a photodetector and can be determined via the equation *R* = *I*_ph_/(*P*_in_×*S*), where *I*_ph_ is the photocurrent, *I*_ph_ = *I*_illumination_ − *I*_dark_, *S* is the active illuminated area, and *P*_in_ is the light power density. *S* and *P*_in_ are 4.5 × 10^−4^ cm^−2^ and 2 mW cm^−2^ in our measurement for the xenon lamp illumination, respectively. As shown in Figure 2c, an initial photocurrent of 0.27 nA and a responsivity of 0.3 mA W^−1^ are determined at an initial pressure of 1.2 GPa. The photocurrent density *J*_ph_ of Cs_3_Bi_2_I_9_ was derived from Figure 2c using the equation *J*_ph_ = *I*_ph_/*S*, as illustrated in Figure 2d. Initially, *I*_ph_ slowly increases upon compression and reaches 2.7 nA (1 mA W^−1^ for *R*) at 9.5 GPa, which is ten times that at 1.2 GPa. Subsequently, *I*_ph_ rapidly increases with further increasing pressure and reaches ≈18 µA (20 A W^−1^ for *R*) at 26.7 GPa, which is almost five orders of magnitude higher than that at 1.2 GPa. Under higher pressure, photoresponse of Cs_3_Bi_2_I_9_ will weaken and eventually disappear owing to it transform into the metallic state.

Notably, the photoresponse speed is suppressed under higher pressure. Generally, such a slow photoresponse speed is strongly correlated with the photothermal effect or trap states of materials.^[^ ^42^, ^43^ ^]^ The trap states can be neglected owing to the high‐quality Cs_3_Bi_2_I_9_ single‐crystal sample with a low trap density. Thus, the slow photoresponse can be mainly attributed to the photothermal effect. Intriguingly, the photothermal effect includes the bolometric effect and photothermoelectric effect, here refers to the bolometric effect. In addition to the slow response speed, the photoresponse dominated by the bolometric effect cannot be achieved with zero bias.^[^ ^42^, ^44^ ^]^ As a characteristic of light‐induced heating, the photothermoelectric effect is the result of converting the temperature difference Δ*T* generated by local illumination to an electric voltage via the Seebeck effect. The photoresponse properties dominated by the photothermoelectric effect possess a fast and broadband spectral response with self‐driven operation without an external bias.^[^ ^39^, ^44^ ^]^

**Figure 3:**
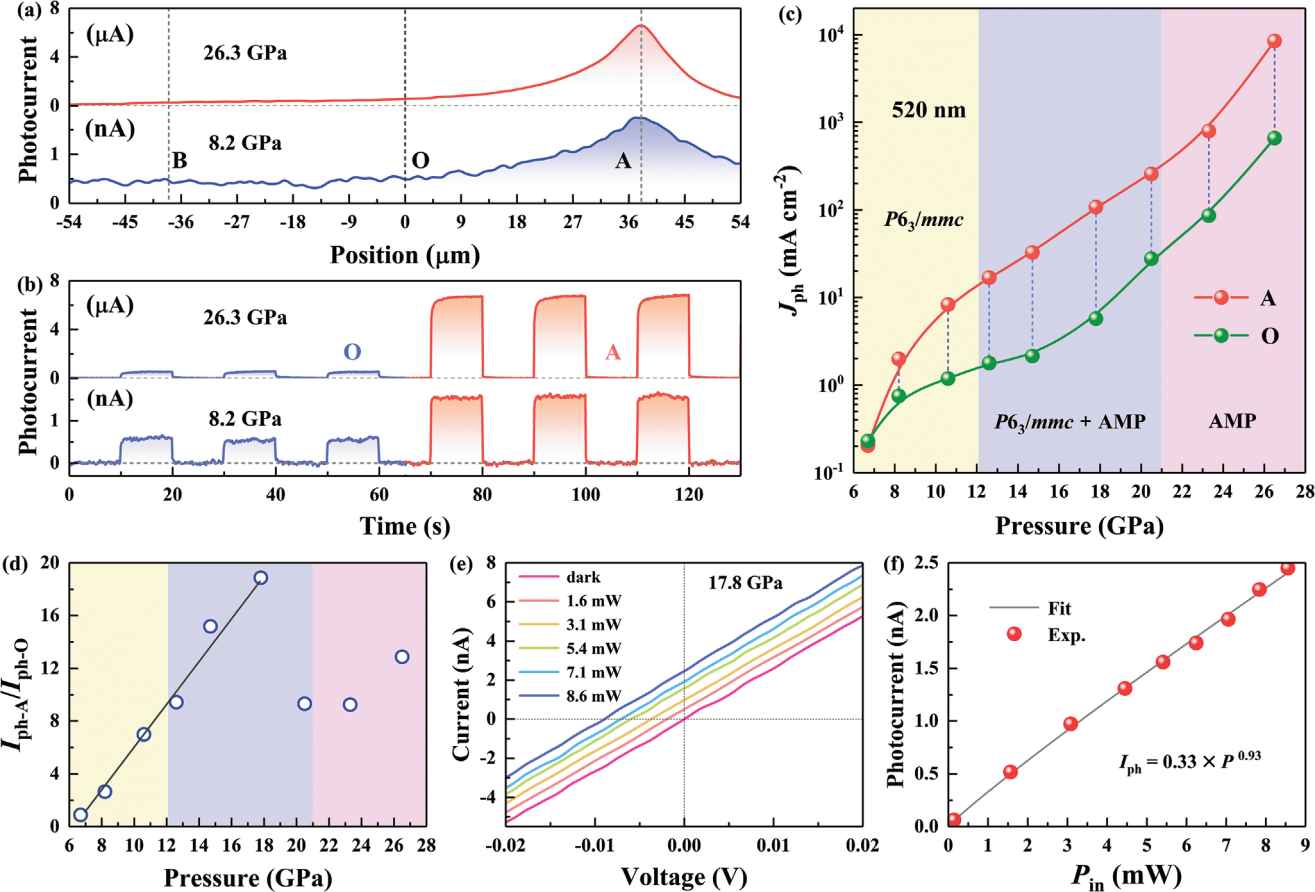
Photoelectric properties of Cs_3_Bi_2_I_9_ with 520 nm laser illumination. a) Position‐dependent photocurrent of Cs_3_Bi_2_I_9_ when the 520 nm laser spot sweeps between the two Pt electrodes under a 10 V bias at selected pressures. b) Variation in photocurrent curves of Cs_3_Bi_2_I_9_ with 520 nm laser illumination at positions A and O under a 10 V bias. c) Pressure‐dependent photocurrent density *J*_ph_ of Cs_3_Bi_2_I_9_ with laser illumination at position A and position O. The data were extracted from (a). d) Pressure dependence of the photocurrent ratio (*I*_ph‐A_/*I*_ph‐O_); *I*_ph‐A_ and *I*_ph‐O_ are the photocurrents with 520 nm laser illumination at positions O and A, respectively. The solid line is only a guide to the eye. e) *I–V* curves of Cs_3_Bi_2_I_9_ under 520 nm laser illumination at position A with different light intensities at 17.8 GPa. f) Light intensity dependence of the photocurrent of Cs_3_Bi_2_I_9_ at 17.8 GPa; the data were extracted from (e).

Considering the obvious photothermal effect of Cs_3_Bi_2_I_9_ under high pressure, the photoresponse can be expected to be dominated by the photothermoelectric mechanism. To further investigate the photothermoelectric effect of Cs_3_Bi_2_I_9_ upon compression, the device was illuminated by local laser illumination with a spot diameter of approximately 10 µm. When the pressure is below 6.7 GPa, the photoresponse of Cs_3_Bi_2_I_9_ with 520 nm laser illumination under a 10 V bias is too weak to be detected because it is lower than the lower resolution limit of the experimental equipment (Figure S3a, Supporting Information). As shown in **Figure** **3**a, the obvious position dependence of the photocurrent indicates the different photoresponse generation mechanisms. The photoconductive effect exists when the incident photon energy is greater than the optical bandgap. The photothermal effect can be negligible owing to the fast photoswitching state of Cs_3_Bi_2_I_9_ whether the laser spot is at position A or position O (Figure 3b). Position O has no photothermoelectric effect because it is at the center of the device and a temperature gradient cannot be formed between the two electrodes. Thus, the photocurrent with laser illumination at position O is contributed mostly by the photoconductive mechanism. Obviously, the photocurrent monotonically increases as the laser spot is swept from position O to the Pt electrode. The enhancement of the photocurrent can be mainly attributed to the photothermoelectric effect. The maximum photocurrent occurs at position A, which is near the Cs_3_Bi_2_I_9_/Pt interface.

The photocurrents of Cs_3_Bi_2_I_9_ with laser illumination at positions A and O upon further increasing the pressure were further investigated. As shown in Figure 3b,c, with increasing pressure, the photocurrent density (*J*_ph_) exhibits a significant increase at different rates under the laser spot at position A and position O. To obtain further insight into the difference in the photocurrents with laser illumination at position A and position O, the variations in the photocurrent ratio *I*_ph‐A_/*I*_ph‐O_ as a function of pressure are illustrated in Figure 3d. The photocurrent ratio monotonically increases from 0.9 to 18.9 until the pressure reaches 17.8 GPa. When the pressure exceeds 17.8 GPa, the photocurrent ratio is almost unchanged and converges to a stable value, which indicates that the photocurrent contributed by the photothermoelectric effect increases with increasing pressure to 17.8 GPa and then tends to stabilize. Intriguingly, the fast, stable, and high photoresponse with 520 nm laser illumination at position A is the result of the synergy of the photoconductive and photothermoelectric effects.

The self‐driven photoresponse is illustrated in Figures S4a and S5a, Supporting Information. A photocurrent of 0.2 nA under zero bias at 8.2 GPa is detected, which exhibits a successive enhancement upon further compression to 17.8 GPa and then tends to be stable around 2 nA under higher pressure (Figure S4b, Supporting Information). This is consistent with the trend of the photocurrent ratio under high pressure illustrated in Figure 3d. And because of the photoresponse does not only happens at contacts region (Figure S4a,c, Supporting Information), the Photo‐Dember (PD) effect can be excluded owing to it only occurs at the overlapping region of Cs_3_Bi_2_I_9_‐Pt where breaks the electron‐hole symmetry. The incident light intensity dependence of the photoelectric properties at 17.8 GPa was further evaluated. As shown in Figure 3e, the *I–V* curves shift upward nearly parallelly upon increasing the illumination intensity, which corresponds well with the photothermoelectric characteristics of the device.^[^ ^39^, ^45^ ^]^ With increasing incident light power, the photocurrent exhibits a monotonic increase, and the dependence can be fitted via the general power equation *I*_ph_ = 0.33×*P* ^0.93^ (Figure 3f).

The photoresponse properties of Cs_3_Bi_2_I_9_ were systematically evaluated under different laser wavelengths from 635 to 1650 nm. At ambient pressure, a photoresponse contributed by the photoconductive effect with 635 nm laser illumination is impossible owing to the photon energy being below the bandgap of 2.0 eV. The photoresponse generated by the photothermoelectric mechanism exhibits a broadband spectral response and is not limited by the bandgap of photoelectric materials. However, it is too weak to be detected before the pressure reaches 6.7 GPa. As illustrated in Figure S6, Supporting Information, the photocurrents at position A and position O are both approximately 0.26 nA (*J*_ph_ of 0.33 mA cm^−2^) with 635 nm laser illumination, which indicates that the photothermoelectric effect is very weak and that the photoconductive effect contributes almost all of the photocurrent at a current pressure of 6.7 GPa.^[^ ^42^ ^]^ With further increasing pressure, the photocurrents exhibit rapid enhancement, especially for the laser spot at position A. The photocurrents reach 16.7 and 1.2 µA (*J*_ph_ of 21 210 and 1567 mA cm^−2^) with laser illumination at positions A and O, respectively, which are almost five and four orders of magnitude enhancements in comparison to the initial values at 6.7 GPa, respectively.

**Figure 4:**
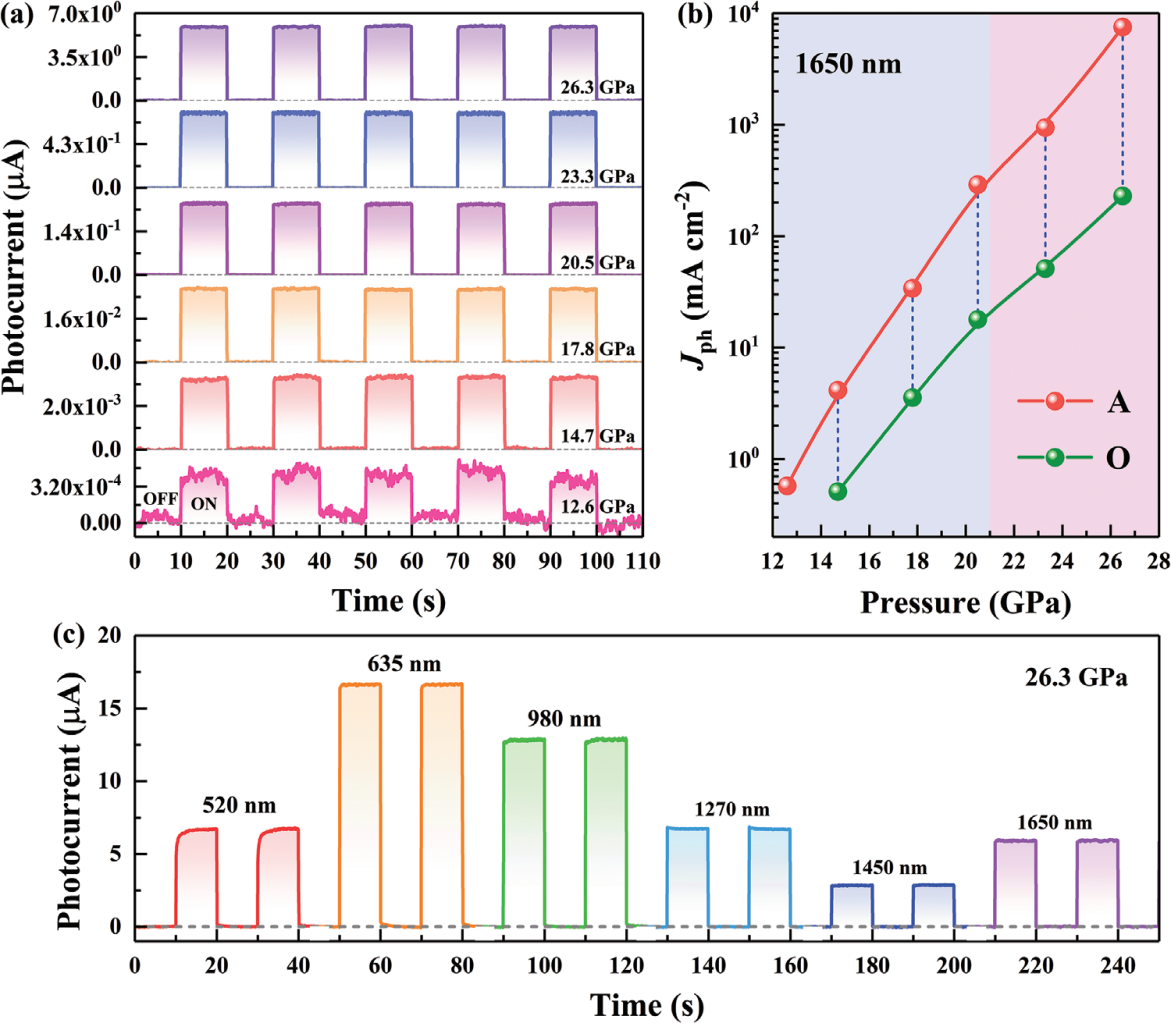
Photoresponse properties of Cs_3_Bi_2_I_9_ under laser illumination of different wavelengths. a) Photocurrent of Cs_3_Bi_2_I_9_ during compression with 1650 nm laser illumination at position A under a 10 V bias. b) Pressure‐dependent photocurrent density *J*_ph_ of Cs_3_Bi_2_I_9_ under 1650 nm laser illumination at positions A and O. The data were extracted from (a). c) Photocurrent of Cs_3_Bi_2_I_9_ under laser illumination of different wavelengths at position A under a 10 V bias at 26.3 GPa.

Upon further compression, the photoelectric spectral response range of Cs_3_Bi_2_I_9_ can be extended to the near‐infrared band and successively generates a photoresponse to 980, 1270, 1450, and 1650 nm laser illumination via the photoconductive effect or photothermoelectric effect at higher pressure (**Figure** **4**a; Figures S7–S9, Supporting Information). For example, photocurrents of 0.45 and 0.41 nA with 1650 nm laser illumination at position A and position O are detected with pressures of 12.6 and 14.7 GPa, respectively (Figure 4a,b), too weak photoresponse even causes curve jitter. As shown in Figure 4a and Figures S7–S9, Supporting Information, the photocurrent exhibit significant enhancement and the curve jitter successively reduced and ultimately disappeared upon further increasing the pressure, with similar change trends to that of the photocurrent with 635 nm laser illumination. Furthermore, Cs_3_Bi_2_I_9_ exhibits a fast photoresponse speed with rise and decay times of ≈95 and 96 ms, respectively. Under near‐infrared illumination, the photoresponse speed is almost not suppressed and is maintained upon increasing the pressure, while the rise time with 520 nm laser illumination was increasing obviously (Figure S17b and Table S1, Supporting Information), which may be caused by the higher photothermal conversion efficiency of Cs_3_Bi_2_I_9_ to 520 nm laser illumination and result in the more obvious bolometric effect under high pressure. As illustrated in Figure 4c, photocurrents of 6.7, 16.7, 12.9, 6.7, 2.8, and 5.9 µA (*J*_ph_ of 8.5, 21.2, 16.4, 8.5, 3.6, and 7.5 A cm^−2^) under laser illumination with different wavelengths of 520, 635, 980, 1270, 1450, and 1650 nm are achieved when the pressure reaches 26.3 GPa. Impressively, Cs_3_Bi_2_I_9_ delivers a stable, fast, reproducible and broadband photoresponse from 520 nm to the optical communication waveband (1650 nm) with a responsivity *R* of 6.81 mA W^−1^ at 1650 nm (Table S1, Supporting Information). Such an ultrabroadband photoresponse is representative and innovative in numerous perovskite materials (Table S2, Supporting Information)^[^ ^9^, ^10^ ^]^ and can even be comparable to that of the conventional amorphous materials in previous work (Table S3, Supporting Information).

**Figure 5:**
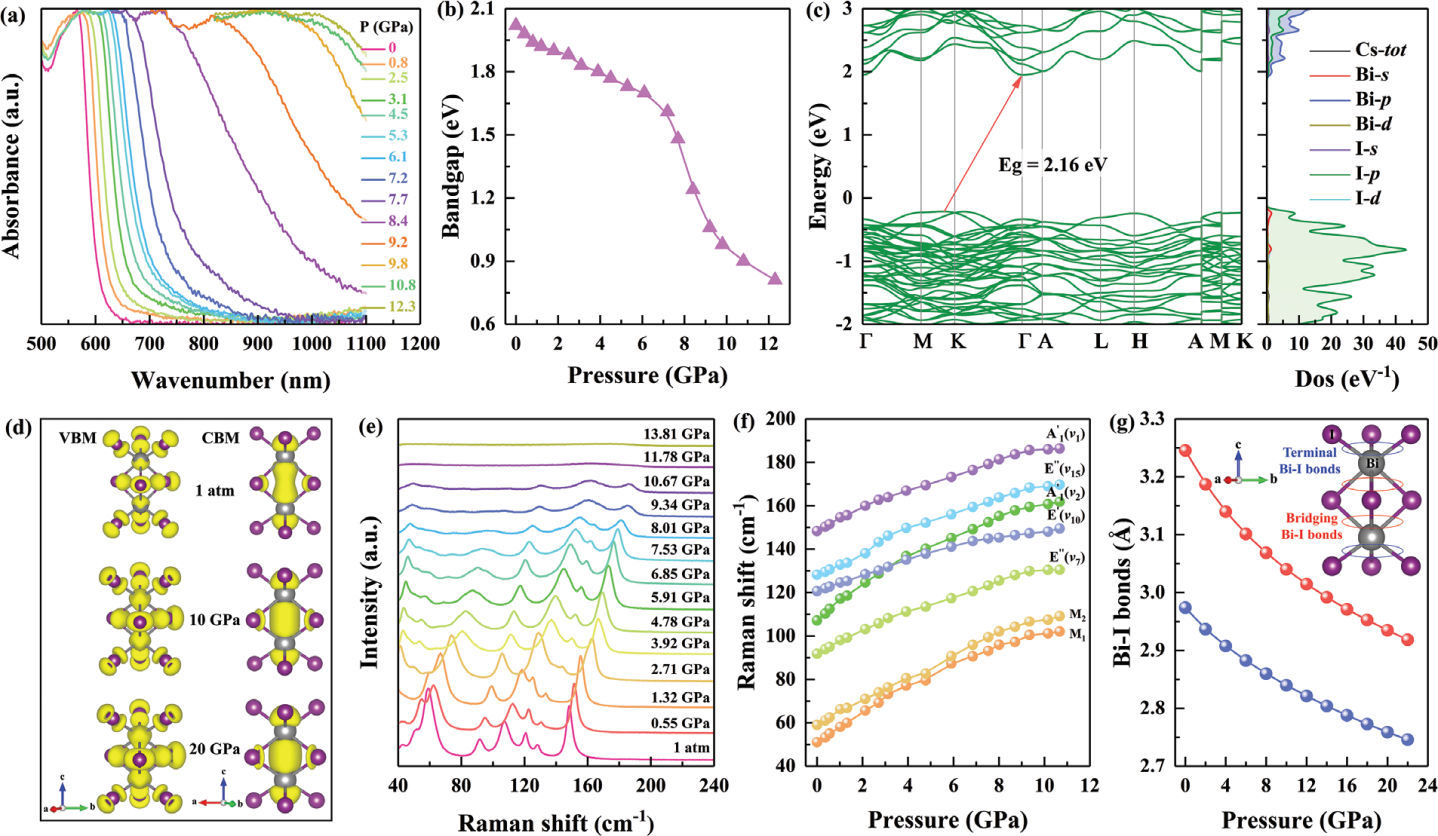
Optical properties and electronic structure. a) Optical absorption spectrum of Cs_3_Bi_2_I_9_ at selected pressures. b) Variations in the optical bandgap of Cs_3_Bi_2_I_9_ as a function of pressure. c) Electronic band structure and partial density of states of Cs_3_Bi_2_I_9_ at ambient pressure. d) Calculated charge distribution of the VBM and CBM of Cs_3_Bi_2_I_9_ at selected pressures. e) Raman spectra of Cs_3_Bi_2_I_9_ at selected pressures. f) Variations in the Raman shift of Cs_3_Bi_2_I_9_ as a function of pressure. g) Pressure dependence of the calculated Bi—I bond length of Cs_3_Bi_2_I_9_.

The optical absorption spectrum and bandgap are critical characteristics for determining practical applications in the photoelectric field. **Figure** **5**a illustrates the pressure dependence of the optical absorption spectrum of Cs_3_Bi_2_I_9_ up to 12.3 GPa. Initially, the absorption edge slowly redshifts with compression from ambient pressure to 7.7 GPa, and then, the absorption edge dramatically redshifts upon further compression. Correspondingly, the optical bandgap is derived from the optical absorption spectra illustrated in Figure 5b, which suffers a slow reduction from 2.0 to 1.5 eV with increasing pressure from ambient pressure to 7.7 GPa and then rapidly decreases to ≈0.8 eV with increasing pressure to 12.3 GPa. This is correlated with the evolutionary trend of the pressure‐dependent photocurrent density and resistance, as illustrated in Figure 2d and Figure S11b, Supporting Information. Intriguingly, the reduction of the bandgap would favor a continuous intrinsic absorption threshold increase and generate a photoresponse to incident light with longer wavelengths via the photoconductive mechanism, resulting in extension of the spectral response range from the visible light band to the optical communication waveband.

As shown in Figure 5c, the calculated band structure reveals that Cs_3_Bi_2_I_9_ is an indirect bandgap semiconductor with a bandgap of 2.16 eV, which well matches the experimental result. According to the calculated electronic density of states (DOS), the valence band maximum (VBM) mainly consists of the I‐5p orbital with a small contribution of the Bi‐6s orbital, whereas the conduction band minimum (CBM) originates from the I‐5p orbital and Bi‐6p orbital in each [Bi_2_I_9_]^3−^ cluster (Figure 5d). With increasing pressure, the VBM and CBM continuously move to the Fermi level, ultimately causing the bandgap reduction (Figure S12, Supporting Information). Thus, the optical bandgap and photoelectric property evolution of Cs_3_Bi_2_I_9_ with pressure is dominated by the dynamic behavior of the [Bi_2_I_9_]^3−^dioctahedral cluster.

To understand the dynamic behavior of the [Bi_2_I_9_]^3−^ cluster under high pressure, the variations in the Raman spectrum with pressure were investigated, as shown in Figure 5e. The dominant vibrational modes originate from the [Bi_2_I_9_]^3−^ cluster, and the dependence of the Raman shift on pressure reveals the Bi—I interactions in the [Bi_2_I_9_]^3−^ octahedra.^[^ ^29^ ^]^ The pressure dependence of Raman shift is closely related to the variation of interatomic interactions, which is a powerful tool for understanding the charge‐transfer and variation of photoelectric properties.^[^ ^26^, ^28^, ^46^ ^]^ As shown in Figure 5f, with increasing pressure, all vibrational modes successively shift to higher frequencies and enhance electron localization function (ELF) accumulation between Bi and I atoms in each [Bi_2_I_9_]^3−^ octahedron (Figure S14, Supporting Information), which can be attributed to the Bi—I bond shortening and significant enhancement in Bi—I interactions. The Raman modes shift to higher frequencies with different rates indicate the different pressure sensitivity of interatomic interaction. In particular, the bridging Bi—I symmetric stretching mode A_1_^1^(*v*_2_) rapidly blueshifts and surpasses the terminal Bi—I antisymmetric stretching mode E′(*v*_10_) with the pressure reaching ≈3 GPa,^[^ ^29^ ^]^ which is associated with the more pronounced shrinkage of bridging Bi—I bonds than of terminal Bi—I bonds in Figure 5g. The shortening of Bi—I bond lengths and decreased bridging Bi—I—Bi angle (Figure S15, Supporting Information) would promote enhanced atomic orbital overlap between I and Bi (Figure 5d), as eventually reflected in the significant enhancement in photoelectric properties.

With further increasing pressure, almost all Raman peaks broaden at 9.3 GPa and disappear when the pressure exceeds ≈10.7 GPa, indicating that the Cs_3_Bi_2_I_9_ starts to transform into the amorphous state owing to the pressure‐induced unordered arrangement of the [Bi_2_I_9_]^3−^ octahedra.^[^ ^29^ ^]^ Intriguingly, the pressure‐induced amorphization of Cs_3_Bi_2_I_9_ would not restrict the enhanced atomic orbital overlap within the [Bi_2_I_9_]^3−^ octahedra upon further compression,^[^ ^22^, ^29^ ^]^ indicating successive enhancement of photoelectric properties in amorphous Cs_3_Bi_2_I_9_. Generally, amorphization may result in the increase B—X (B = Pb, Sn, etc.; X = Cl, Br, I) bond distance or B—X—B bond angles, which would decrease the degree of orbital overlap, ultimately result in the bandgap and resistance increase, and the photocurrent is suppressed.^[^ ^22^, ^24^, ^47^, ^48^ ^]^ Typical examples include MAPbBr_3_,^[^ ^24^ ^]^ MAPbI_3_,^[^ ^47^ ^]^ and FAPbBr_3_.^[^ ^48^ ^]^ Thus, significant enhancement of photoresponse in amorphous Cs_3_Bi_2_I_9_ is precious and unachieved in previous literatures. Impressively, the pressure‐induced amorphization would result in a lower lattice thermal conductivity,^[^ ^32^, ^33^, ^34^, ^35^, ^36^ ^]^ which would promote enhancement of photoelectric properties of Cs_3_Bi_2_I_9_ dominated by the photothermoelectric mechanism, as evidenced by the more rapid enhancement in the photocurrent with laser illumination at position A than at position O (Figure S16, Supporting Information).

## 3 Conclusion

In conclusion, we systematically investigated the photoelectric properties of the halide perovskite Cs_3_Bi_2_I_9_ through comprehensive in situ high‐pressure probes and theoretical calculations. Under large‐scale xenon lamp illumination, a dramatic enhancement in the photocurrent by almost five orders of magnitude occurs in the pressure‐induced amorphization process. Intriguingly, a stable, high‐gain, pressure‐tunable, and broadband photoresponse from the visible to optical communication waveband (1650 nm) with a responsivity of 6.81 mA W^−1^ at 1650 nm is achieved upon further successive compression, which originates from the comprehensive result of the photoconductive and photothermoelectric mechanisms. Extensive spectroscopy characterizations and theoretical calculations reveal that the tunable and exceptional photoelectric properties of Cs_3_Bi_2_I_9_ are associated with a dramatic increase in Bi—I interactions within the [BiI_6_]^3−^ cluster, which would enhance the degree of orbital overlap, even in the amorphous structure form. Ultimately, this causes bandgap narrowing and significantly enhanced photoelectric properties in the halide perovskite Cs_3_Bi_2_I_9_. Our findings signify that amorphous Cs_3_Bi_2_I_9_ possesses immense potential for photoelectric applications, which opens up a new avenue for further investigating the unexplored properties and applications of amorphous perovskites.

## 4 Experimental Section

### Sample Synthesis and Characterization

The inverse temperature crystallization method was used to grow high‐quality, cm‐scale Cs_3_Bi_2_I_9_ single crystals using a procedure described in previous work.^[^ ^14^ ^]^ The crystal structure of the Cs_3_Bi_2_I_9_ sample was determined by XRD (DX‐2700BH) with Cu*kα* radiation (*λ* = 1.5406 Å). The PL spectrum was obtained via a Renishaw inVia notch filter spectrometer with a 514.5 nm excitation laser under ambient conditions.

### High‐Pressure In Situ Photoelectric Property Measurements

High‐pressure in situ measurements of Cs_3_Bi_2_I_9_ were performed in a symmetrical DAC with a pair of anvil culets of 300 µm. A T301 stainless steel gasket was preindented to ≈50 µm in thickness, and a hole with a suitable diameter was drilled via a laser drilling system to serve as the sample chamber. Photoelectric property measurements were carried out via the two‐probe method. A mixture of epoxy and boron nitride (c‐BN) powder was loaded into the DAC chamber and then firmly compressed to achieve electrical insulation between the metal electrodes and steel gasket. A layer of crushed Cs_3_Bi_2_I_9_ single crystal sample with a thickness of ≈30 µm and size of ≈300 µm was placed on the c‐BN insulating layer. Pt possesses excellent ductility, the Seebeck coefficient (−1.87 µV K^−1^) was much lower than that of the sample, and variations in Seebeck coefficient and resistance of Pt with pressure was relatively small, which hardly interfered with the measurement results. Thus two platinum sheets served as electrodes to contact the Cs_3_Bi_2_I_9_ sample in the DAC chamber. No pressure‐transmitting medium was used. The photoelectric property measurements under laser illumination were conducted using a high‐precision photocurrent scanning test microscope system (MStarter 200) combined with a combination of sourcemeters (Keithley 6482), and lasers of different wavelengths (520, 635, 980, 1270, 1450, and 1650 nm) were used as the light illumination source. The *I–t* and *I–V* data with xenon lamp (CEL‐HXF300) illumination were recorded using a combination of sourcemeters (Keithley 2461). The incident light power was obtained via a probe of the power meter placed under the one‐side diamond anvil on the original measurement position. The area of the laser spot with diameter of 10 µm was the active area to obtain the responsivity under laser illumination.

### Raman Spectroscopy and Optical Absorption Spectroscopy

Raman spectra of Cs_3_Bi_2_I_9_ were recorded on a LabRAM HR Evolution Raman spectrometer (*λ* = 785 nm). No pressure‐transmitting medium was used in the high‐pressure Raman scattering experiments. Optical absorption spectra were acquired using an *i*HR‐320 spectrometer. A KBr film was loaded into the DAC chamber as the pressure‐transmitting medium and the background. The optical absorption spectra were calculated via the expression *α* = log_10_(*T*_ref_/*T*_s_), where *T*_ref_ and *T*_s_ are the transmittances of KBr and Cs_3_Bi_2_I_9_, respectively.

### Theoretical Calculations

The crystal structure and electronic property calculations were implemented using the Vienna Ab Initio Simulation Package (VASP).^[^ ^49^, ^50^ ^]^ The Perdew−Burke−Ernzerhof (PBE) functional within the generalized gradient approximation (GGA)^[^ ^51^ ^]^ was selected to describe the exchange‐correlation effects, and the projector augmented wave (PAW) method^[^ ^52^, ^53^ ^]^ was used in this work. Cs_3_Bi_2_I_9_ possesses a 0D molecular salt crystal structure, the van der Waals (vdWs) dispersion correction was required. Combined with literatures and calculation results of different methods (Table S4, Supporting Information),^[^ ^14^ ^]^ the DFT‐D3 correction^[^ ^54^, ^55^ ^]^ was used to describe the vdWs interaction in the crystal structure of Cs_3_Bi_2_I_9_. The plane wave cutoff energy was set as 600 eV, and a Monkhorst−Pack mesh of 5×5×2 *k*‐points was applied for the structure optimizations and self‐consistent calculations. The convergence thresholds between two optimization cycles for the energy change and maximum force were within 10^−7^ eV and 0.01 eV Å^−1^, respectively. The band structure and partial density of states calculations were postprocessed via the VASPKIT package.^[^ ^56^ ^]^

## Conflict of Interest

The authors declare no conflict of interest.

## Supporting information

Supporting Information

## Data Availability

The data that support the findings of this study are available from the corresponding author upon reasonable request.
