# Supplementary material for: Pressure‐Tuning Photothermal Synergy to Optimize the Photoelectronic Properties in Amorphous Halide Perovskite Cs3Bi2I9
Source: Adv Sci (Weinh). 2022 Dec 29;10(6):2205837. doi: 10.1002/advs.202205837 (PMC9951572; doi:10.1002/advs.202205837)
Supplement: Supplementary file 1 — Supporting Information [file ADVS-10-2205837-s001.pdf]

ADVANCED SCIENCE
Open Access

Supporting Information

for *Adv. Sci.*, DOI 10.1002/advs.202205837

Pressure-Tuning Photothermal Synergy to Optimize the Photoelectronic Properties in Amorphous Halide Perovskite $Cs_3Bi_2I_9$

Zonglun Li, Binxia Jia, Sixue Fang, Quanjun Li, Fuyu Tian, Haiyan Li, Ran Liu, Yucheng Liu*, Lijun Zhang, Shengzhong (Frank) Liu and Bingbing Liu**

Supplementary Information

Pressure-Tuning Photothermal Synergy to Optimize the Photoelectronic Properties in Amorphous Halide Perovskite $Cs_3Bi_2I_9$

Zonglun Li, Binxia Jia , Sixue Fang, Quanjun Li, Fuyu Tian, Haiyan Li, Ran Liu, Yucheng Liu*, Lijun Zhang, Shengzhong (Frank) Liu, Bingbing Liu**

Z. Li, S. Fang, Q. Li, H. Li, R. Liu, B. Liu

State Key Laboratory of Superhard Materials,

Jilin University,

Changchun 130012, China

E-mail: liquanjun@jlu.edu.cn; liubb@jlu.edu.cn

B. Jia, Y. Liu, S. (F.) Liu

Key Laboratory of Applied Surface and Colloid Chemistry, National Ministry of Education,

Shaanxi Engineering Lab for Advanced Energy Technology,

School of Materials Science and Engineering,

Shaanxi Normal University,

Xi'an, 710119, China.

E-mail: liuyc@snnu.edu.cn

F. Tian, L. Zhang

Key Laboratory of Automobile Materials of MOE and School of Materials Science and Engineering,

Jilin University,

Changchun, 130012, China.

S1: Structural evolution of the XRD pattern of $Cs_3Bi_2I_9$ with pressure.

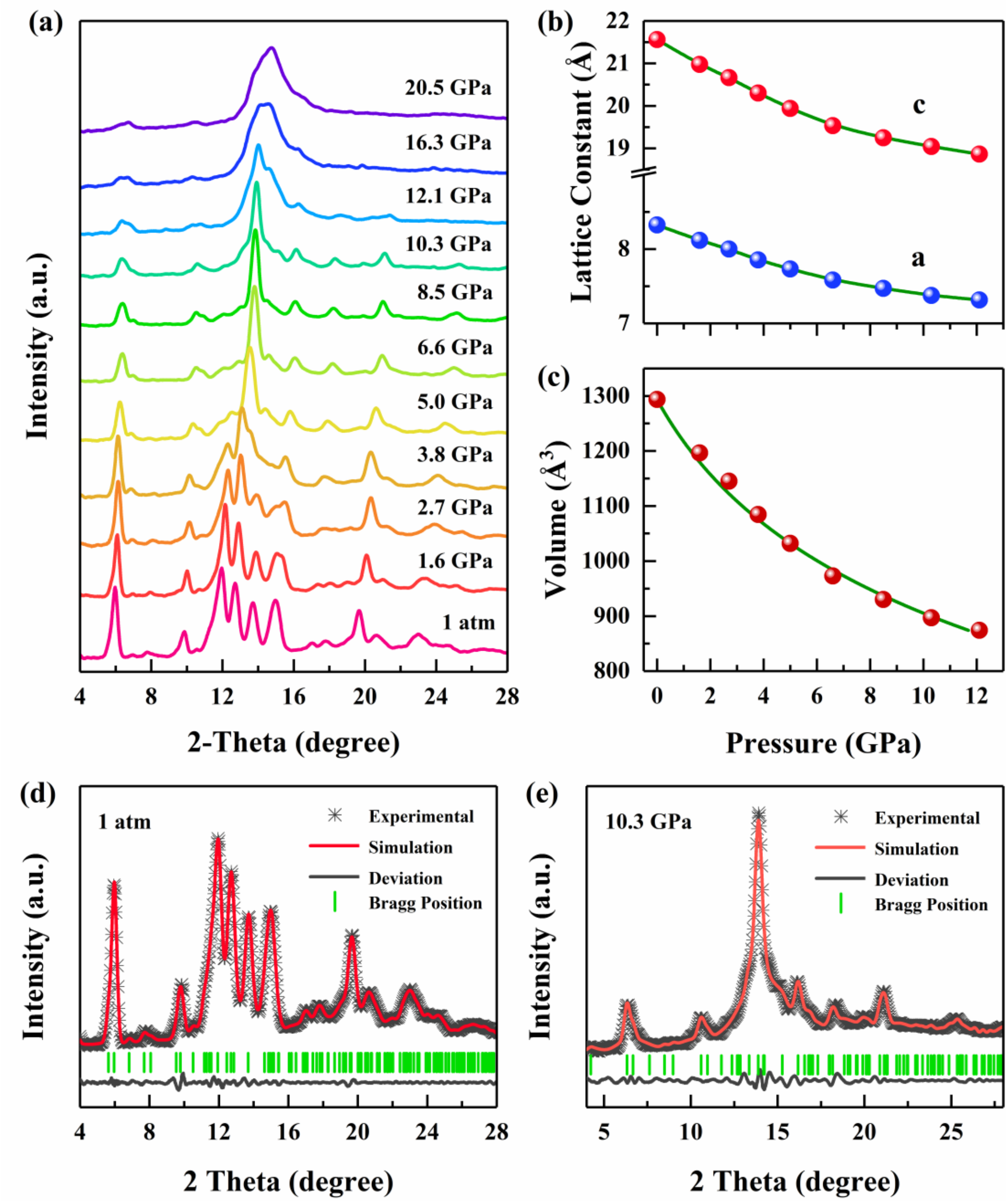

Figure S1. a) Structural evolution of the XRD pattern of $Cs_3Bi_2I_9$ with pressure. b) Variations in the lattice constant as a function of pressure c) and in the volume per formula unit of $Cs_3Bi_2I_9$ as a function of pressure. d-e) Le Bail refinement of the X-ray diffraction (XRD) pattern of $Cs_3Bi_2I_9$ at ambient pressure and 10.3 GPa respectively.

In situ high-pressure XRD experiments were performed using a Rigaku Synergy Custom FR-X diffractometer with Mo K*α* radiation (λ = 0.7099 Å). To ensure the

same experimental conditions as in the photocurrent measurements, no pressure-transmitting medium was used. To obtain the lattice parameters, Le Bail refinements were performed via JANA2006 software.[1]

By fitting the Birch-Murnaghan equation of state, the value of bulk modulus B_0 was determined to be 14.7(6). The Birch-Murnaghan equation is represented by

$$P(V) = \frac{3B_0}{2}\left[\left(\frac{V_0}{V}\right)^{\frac{7}{3}} - \left(\frac{V_0}{V}\right)^{\frac{5}{3}}\right]\{1 + \frac{3}{4}(B' - 4)\left[\left(\frac{V_0}{V}\right)^{\frac{2}{3}} - 1\right]\Bigg\}$$

where B_0 is the bulk modulus, V_0 is the initial volume, V is the volume at pressure, and B' is the derivate of the bulk modulus with respect to pressure.

S2: *I*-*V* curves of $Cs_3Bi_2I_9$ at selected pressures.

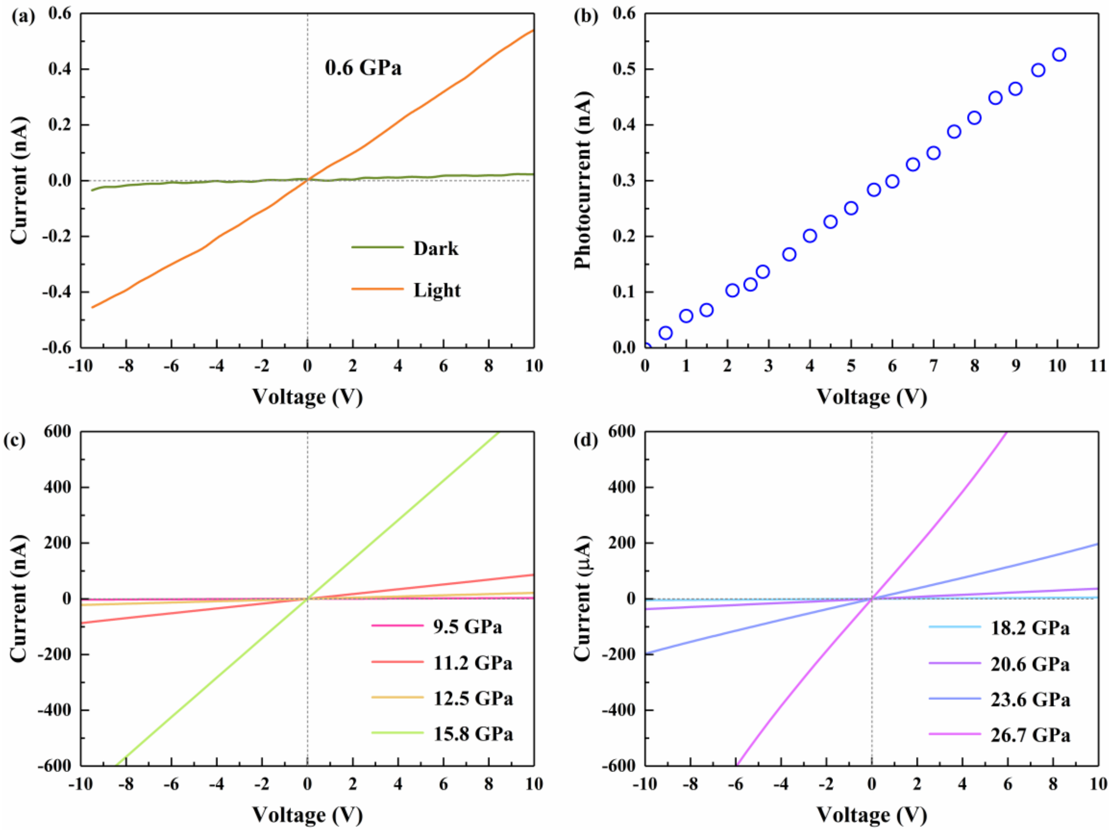

Figure S2. a) *I*-*V* curves of $Cs_3Bi_2I_9$ at an initial pressure of 0.6 GPa with xenon lamp illumination and in the dark. b) Variations in the photocurrent as a function of bias. The data were extracted from Figure S2a. c-d) *I*-*V* curves of $Cs_3Bi_2I_9$ at selected pressures without illumination.

S3: Pressure dependence of the photoelectric properties of $Cs_3Bi_2I_9$ with 520 nm laser illumination under a 10 V bias.

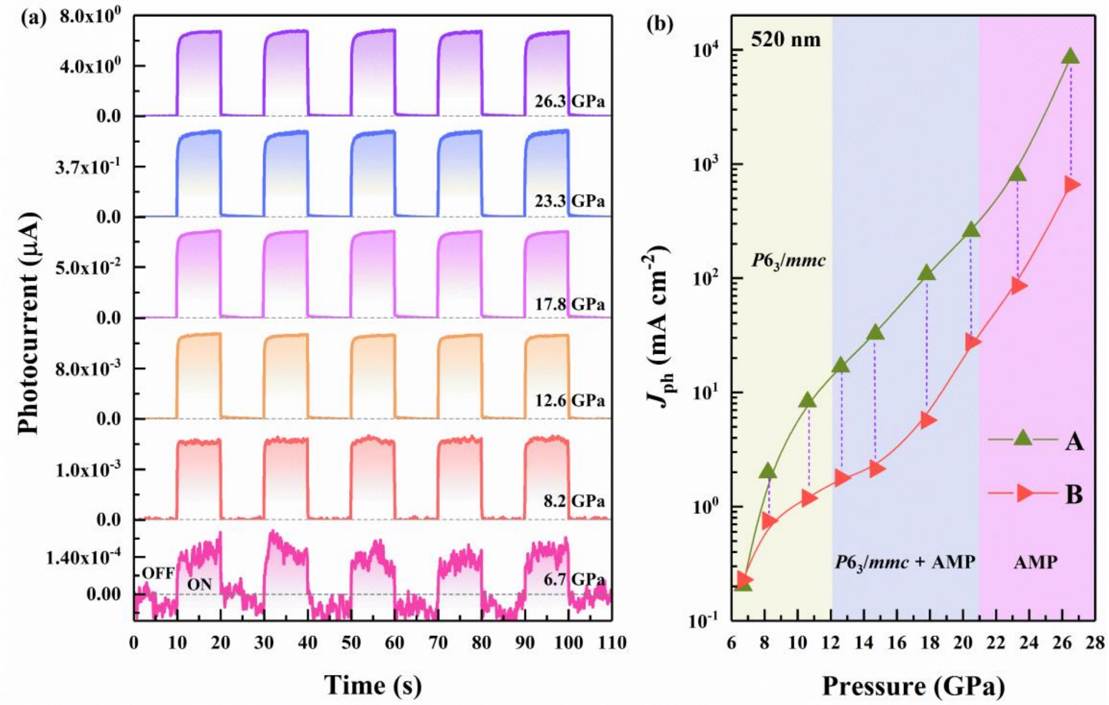

Figure S3. a) Variation in photocurrent curves of $Cs_3Bi_2I_9$ with 520 nm laser illumination at position A under a 10 V bias. b) Pressure dependence of the photocurrent density J_{ph} of $Cs_3Bi_2I_9$ with 520 nm laser illumination at positions A and O. The data were extracted from Figure S3a.

S4: Photothermoelectric characteristics of $Cs_3Bi_2I_9$ under high pressure.

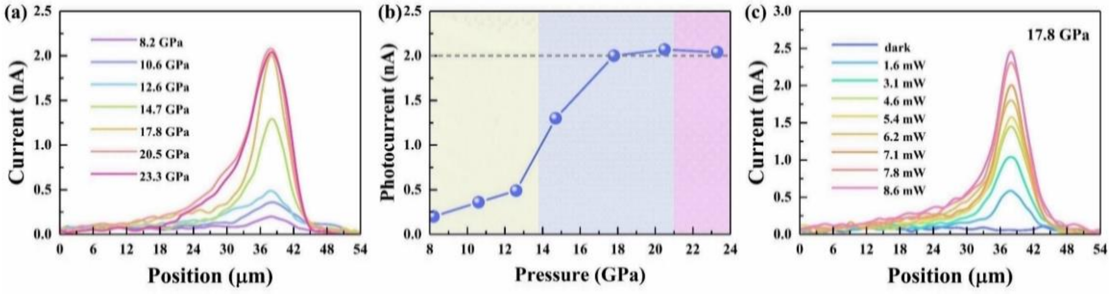

Figure S4. a) Position-dependent photocurrent response of $Cs_3Bi_2I_9$ with the 520 nm laser spot moved from the center of the device (position O) to the *Pt* electrode (position A) under zero bias at selected pressures. b) Pressure dependence of the photocurrent of $Cs_3Bi_2I_9$ with 520 nm laser illumination at position A. The data were extracted from Figure S4a. c) Position-dependent photocurrent response of $Cs_3Bi_2I_9$ when the 520 nm laser spot of different intensities sweeps between the center of the device (position O) and *Pt* electrode (position A) under zero bias at 17.8 GPa.

S5: Photothermoelectric characteristics of $Cs_3Bi_2I_9$ at 17.8 GPa.

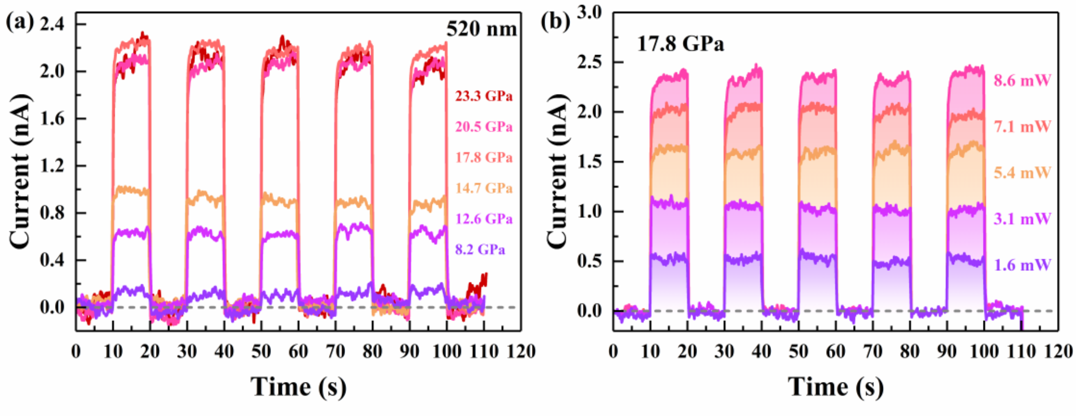

Figure S5. a) Photoresponse of $Cs_3Bi_2I_9$ with 520 nm laser illumination at position A under zero bias at selected pressures. b) Light intensity dependence of the photoresponse of $Cs_3Bi_2I_9$ with 520 nm laser illumination at position A under zero bias at 17.8 GPa.

S6: Pressure dependence of the photoelectric properties of $Cs_3Bi_2I_9$ with 635 nm laser illumination under a 10 V bias.

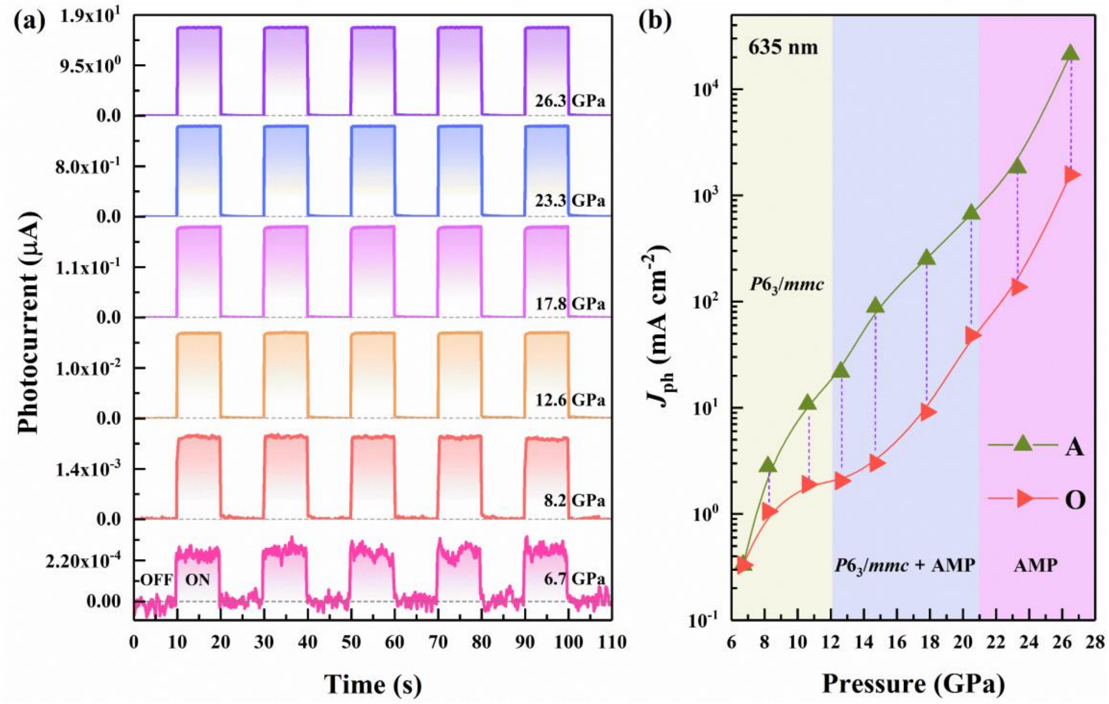

Figure S6. a) Variation in photocurrent curves of $Cs_3Bi_2I_9$ with 635 nm laser illumination at position A under a 10 V bias. b) Pressure dependence of the photocurrent density J_{ph} of $Cs_3Bi_2I_9$ with 635 nm laser illumination at positions A and O. The data were extracted from Figure S6a.

S7: Pressure dependence of the photoelectric properties of $Cs_3Bi_2I_9$ with 980 nm laser illumination under a 10 V bias.

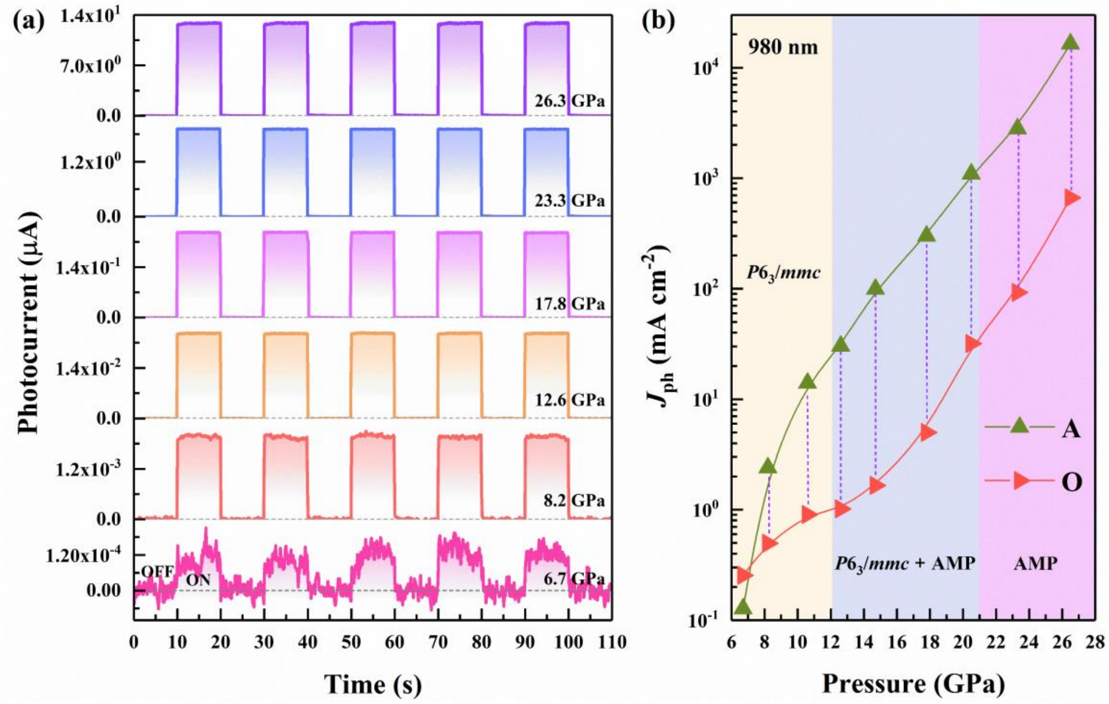

Figure S7. a) Variation in photocurrent curves of $Cs_3Bi_2I_9$ with 980 nm laser illumination at position A under a 10 V bias. b) Pressure dependence of the photocurrent density J_{ph} of $Cs_3Bi_2I_9$ with 980 nm laser illumination at positions A and O. The data were extracted from Figure S7a.

S8: Pressure dependence of the photoelectric properties of $Cs_3Bi_2I_9$ with 1270 nm laser illumination under a 10 V bias.

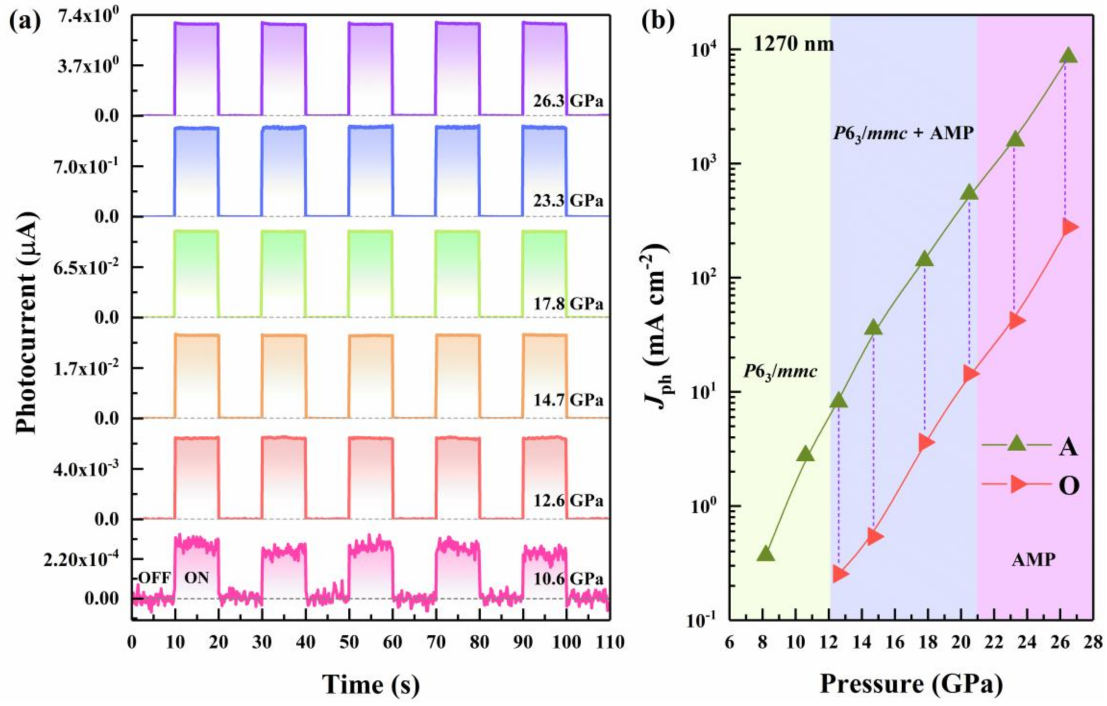

Figure S8. a) Variation in photocurrent curves of $Cs_3Bi_2I_9$ with 1270 nm laser illumination at position A under a 10 V bias. b) Pressure dependence of the photocurrent density J_{ph} of $Cs_3Bi_2I_9$ with 1270 nm laser illumination at positions A and O. The data were extracted from Figure S8a.

S9: Pressure dependence of the photoelectric properties of $Cs_3Bi_2I_9$ with 1450 nm laser illumination under a 10 V bias.

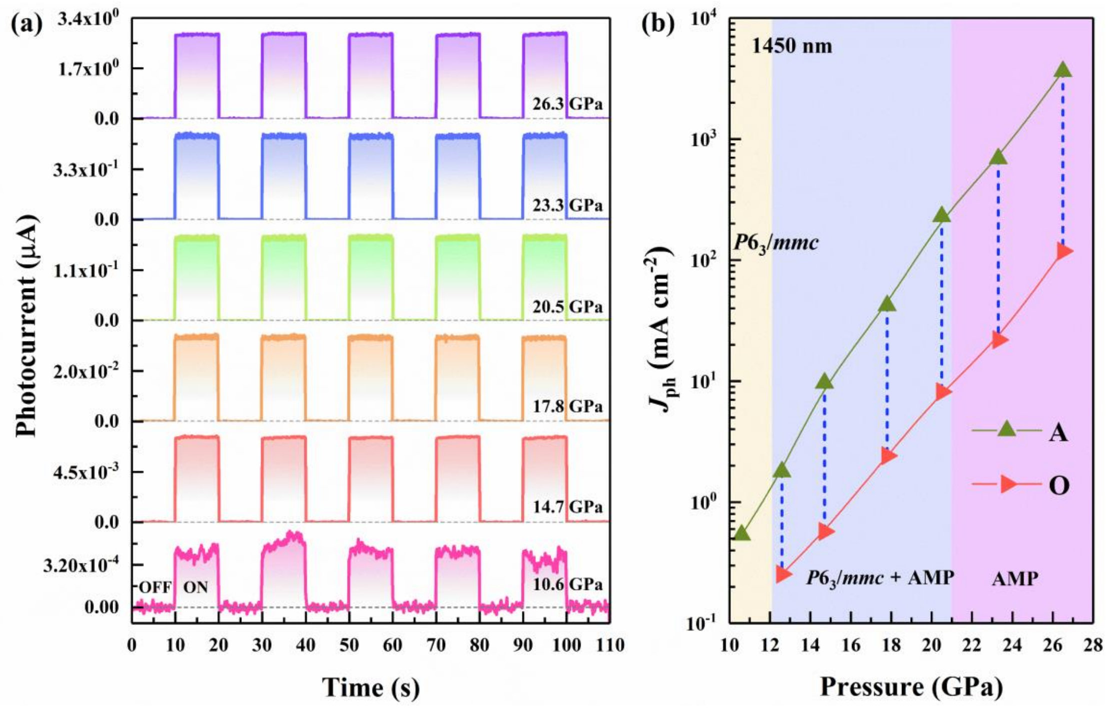

Figure S9. a) Variation in photocurrent curves of $Cs_3Bi_2I_9$ with 1450 nm laser illumination at position A under a 10 V bias. b) Pressure dependence of the photocurrent density J_{ph} of $Cs_3Bi_2I_9$ with 1450 nm laser illumination at positions A and O. The data were extracted from Figure S9a.

S10: External quantum efficiency (*EQE*) of $Cs_3Bi_2I_9$ under high pressure.

The external quantum efficiency (*EQE*) is the ratio of the number of the charge carriers in the photocurrent n_e and the total number of impinging excitation photons n_{total}. It is closely related to the responsivity R, $EQE = R\times(hc/e\lambda)$, c is the light velocity, e is the electron charge, λ is the wavelength of illumination. With similar trends to that of the photocurrent changing with pressure, the *EQE* is significantly enhanced with the increase of pressure, which are almost five or four orders of magnitude enhancements in comparison to the initial values, respectively.

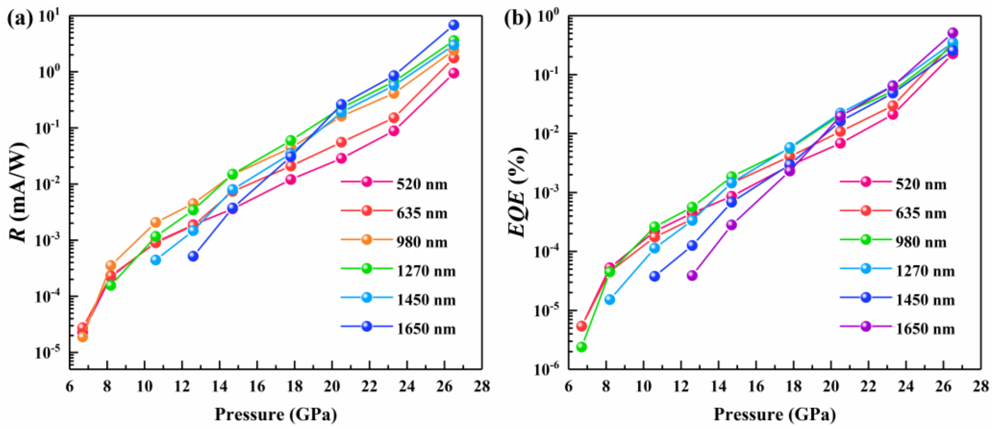

Figure S10 a-b) Variation in R and *EQE* of $Cs_3Bi_2I_9$ with laser illumination at position A under a 10 V bias as a function of pressure.

S11: Current-voltage characteristics and ambient temperature resistance of $Cs_3Bi_2I_9$ under high pressure.

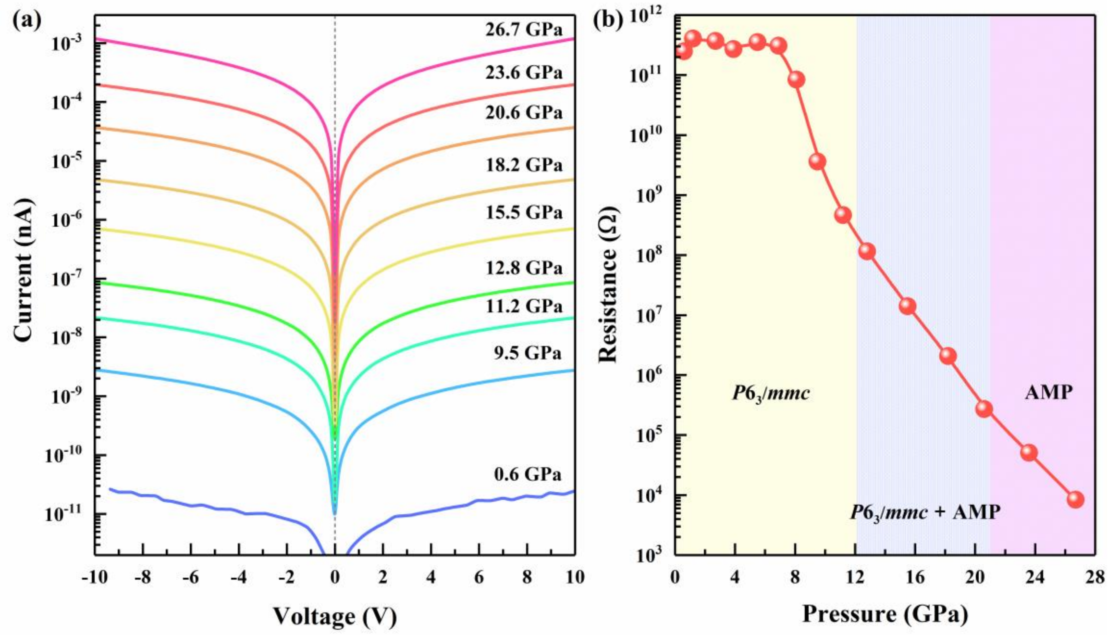

Figure S11. a) Current-voltage characteristics of $Cs_3Bi_2I_9$ at selected pressures. b) Pressure dependence of the ambient temperature resistance of $Cs_3Bi_2I_9$. The data were extracted from the slopes of the *I-V* curves in Figure S11a.

S12: Electronic properties of $Cs_3Bi_2I_9$ under high pressure.

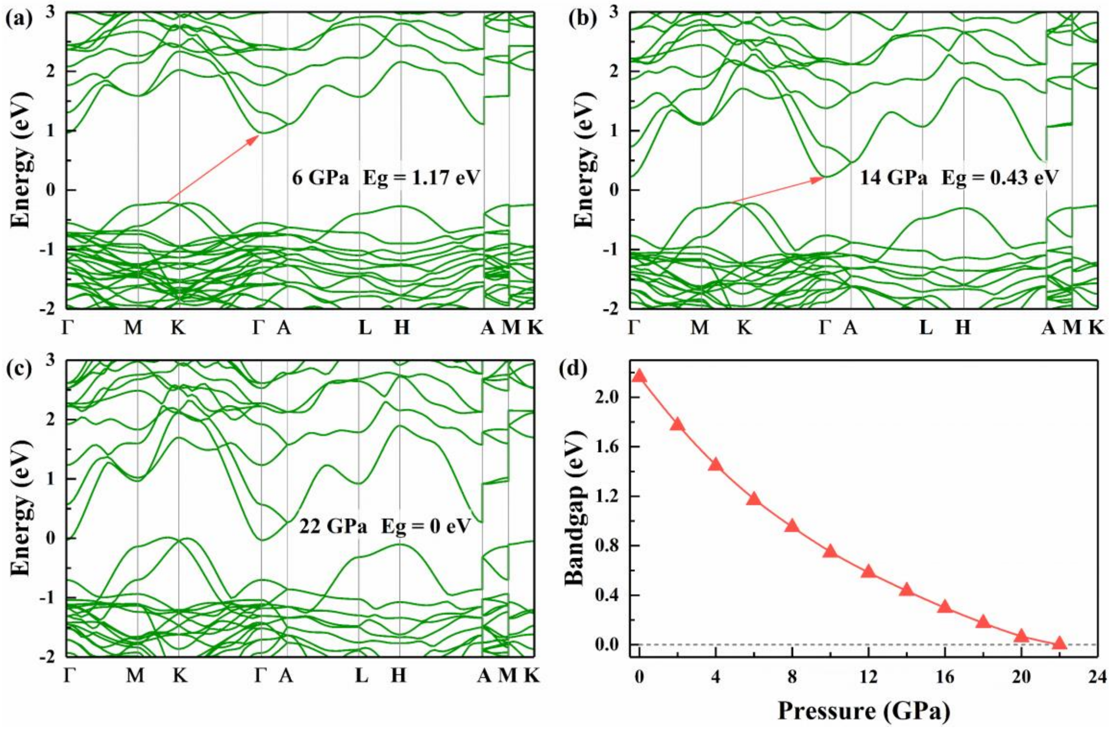

Figure S12. Electronic properties of $Cs_3Bi_2I_9$ under high pressure. a-c) Band structure of $Cs_3Bi_2I_9$ at selected pressures. d) Bandgap of $Cs_3Bi_2I_9$ as a function of pressure.

S13: Raman spectra of $Cs_3Bi_2I_9$.

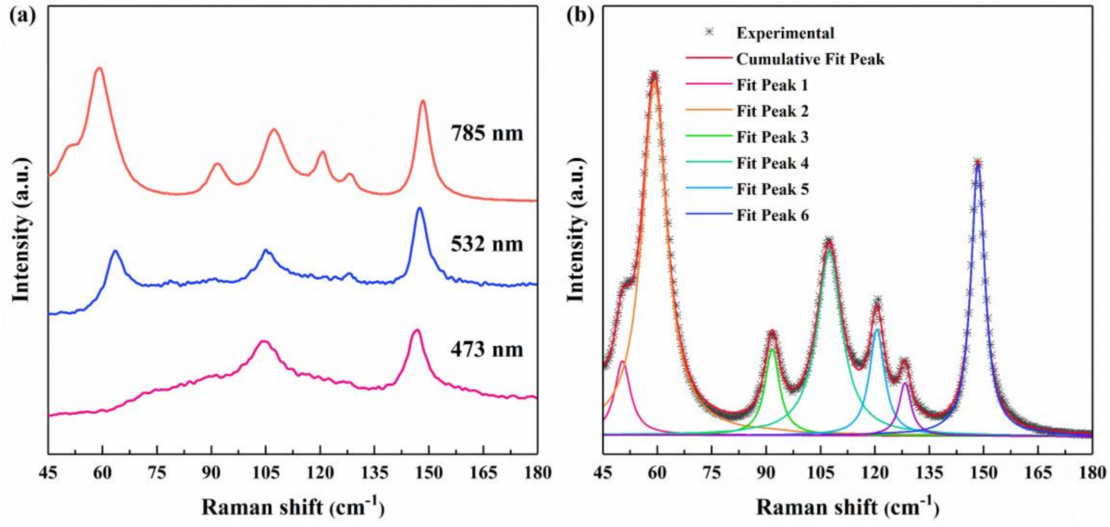

Figure S13 a) Raman spectra of $Cs_3Bi_2I_9$ at ambient pressure with different excitation wavelengths. b) Lorentz fitting for obtaining the wavenumber of each Raman mode at ambient pressure.

S14: ELF of $Cs_3Bi_2I_9$.

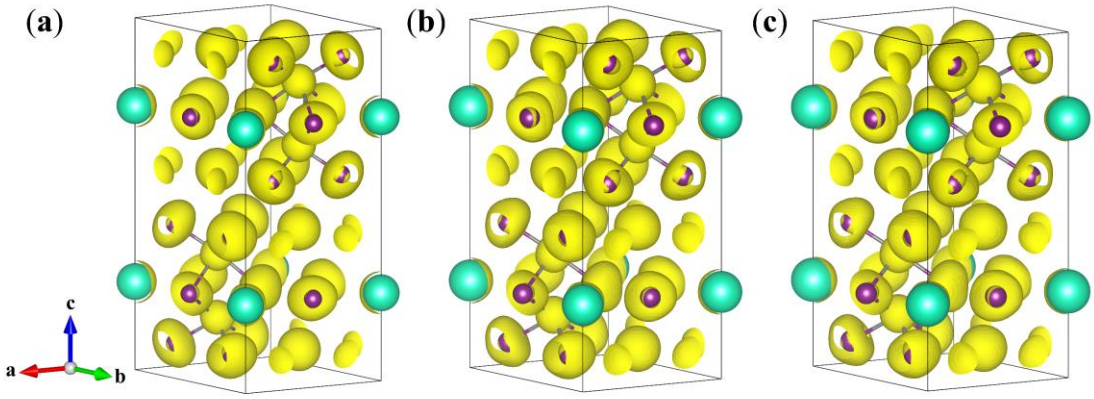

Figure S14. ELF of $Cs_3Bi_2I_9$ with isosurface ELF = 0.80 at selected pressures: a) 1 atm, b) 10 GPa, and c) 20 GPa.

S15: Pressure dependence of the Bi-I-Bi angle of $Cs_3Bi_2I_9$.

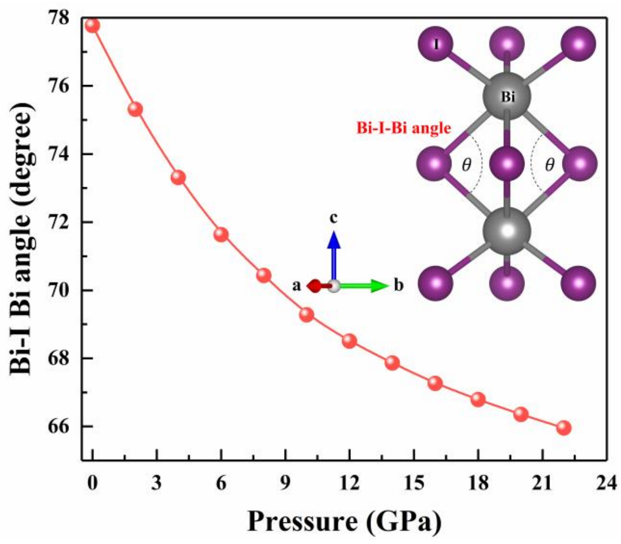

Figure S15. Variations in the calculated Bi-I-Bi angle of $Cs_3Bi_2I_9$ as a function of pressure.

S16: Photocurrent ratio ($I_{ph\text{-}A}/I_{ph\text{-}O}$) with different wavelengths of the laser.

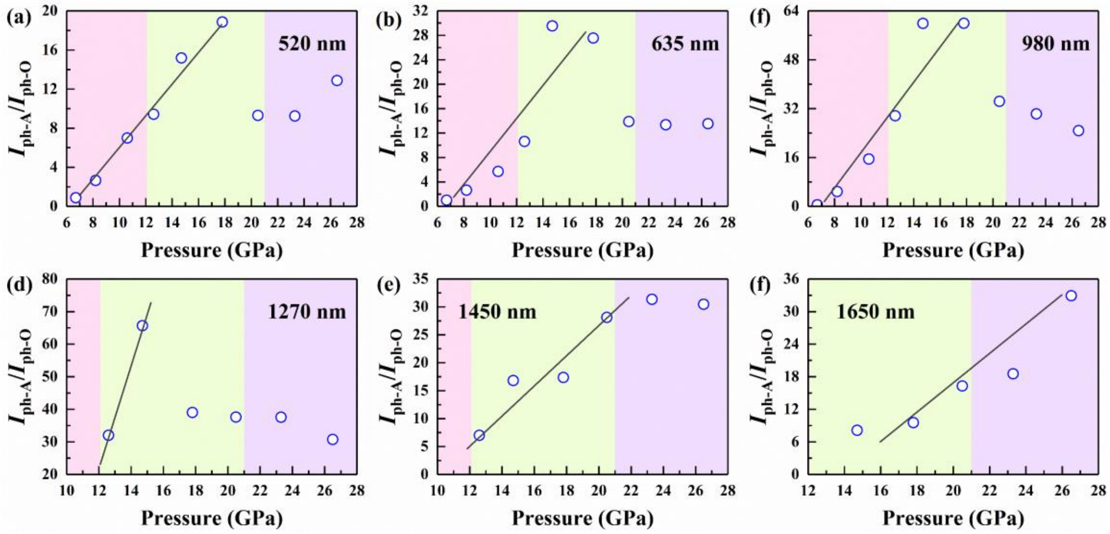

Figure S16. Pressure dependence of the photocurrent ratio ($I_{ph\text{-}A}/I_{ph\text{-}O}$) under laser illumination of different wavelengths: a) 520 nm, b) 635 nm, c) 980 nm, d) 1270 nm, e) 1450 nm, and f) 1650 nm. The increasing trend of the photocurrent ratio with pressure indicates faster increases in the photocurrent with laser illumination at position A than at position O.

S17: Response time of $Cs_3Bi_2I_9$.

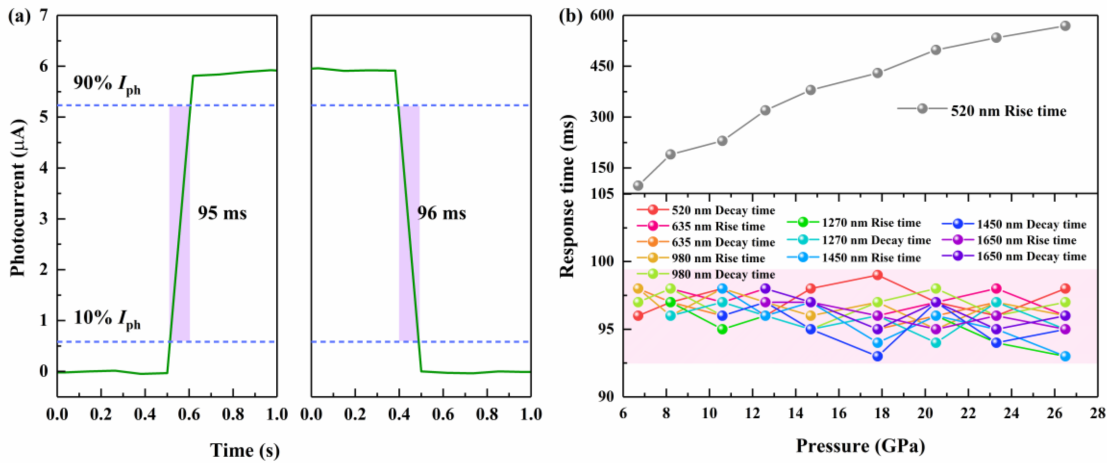

Figure S17. a) Rise time (t_r) and decay time (t_d) of the $Cs_3Bi_2I_9$ device at 26.5 GPa and a 10 V bias under 1650 nm laser illumination. The rise/decay times define the time interval of the response rising (falling) from 10% (90%) to 90% (10%). The fast rise and decay times of the perovskite $Cs_3Bi_2I_9$ are calculated to be 95 and 96 ms, respectively. b) Variations in the rise time and decay time of $Cs_3Bi_2I_9$ device under laser illumination with different wavelengths at position A as a function of pressure.

Table S1. Photoelectric performance comparison of amorphous $Cs_3Bi_2I_9$ under laser illumination of different wavelengths.

Wavelength (nm)	Pressure (GPa)	J_{ph} (mA/cm^2)	Light power (mW)	Responsivity	*EQE* (%)	Response time (ms)
520	6.7	0.20	7.06	0.22 μA/W	5.4×10^{-6}	98/96
520	26.5	8494	7.06	9.45 mA/W	0.23	569/98
635	6.7	0.33	9.47	0.28 μA/W	5.4×10^{-6}	97/98
635	26.5	21210	9.47	1.76 mA/W	0.34	96/96
980	6.7	0.13	5.32	0.19 μA/W	2.4×10^{-6}	98/97
980	26.5	16442	5.32	2.43 mA/W	0.31	96/97
1270	8.2	0.37	1.86	1.56 μA/W	1.5×10^{-5}	97/96
1270	26.5	8535	1.86	3.60 mA/W	0.35	93/95
1450	10.6	0.54	0.95	4.42 μA/W	3.8×10^{-5}	98/96
1450	26.5	3617	0.95	2.99 mA/W	0.26	94/95
1650	12.6	0.57	0.87	5.17 μA/W	3.9×10^{-5}	97/98
1650	26.5	7545	0.87	6.81 mA/W	0.51	95/96

Table S2. Photoelectric performance comparison of amorphous (α) $Cs_3Bi_2I_9$ with some halide perovskite materials.

**Materials**	**Wavelength (nm)**	**Bias (V)**	**Responsivity (mA/W)**	**Response time**	**Ref**
$Cs_3Bi_2I_9$ SCTF*	White LED	2	7.2	247/230 μs	[2]
$Cs_3Bi_2I_9$ SCTF*	450	3	~40	1.5/422 μs	[3]
$Cs_3Bi_2I_9$/Graphene	1064	0	930	31/34 μs	[4]
$Cs_3Bi_2I_9$	450	10	33.1	10.2/37.2 ms	[5]
$CsBi_3I_{10}$/Si	808	1	492.1	73/36 μs	[6]
$CsPbI_3$ NCs*	640	0.5	35	—	[7]
$MAPbI_3$	405	5	15	12/22 ms	[8]
$CsPbI_3$ nanowires	White light	1	6.7	292/234 ms	[9]
$CsPbBr_3$ nanowires	463	5	7.26	10/22 ms	[10]
α-$Cs_3Bi_2I_9$	1650	10	6.81	95/96 ms	This work

SCTF: single-crystalline thin film,
NCs: nanocrystals.

Table S3. Photoelectric performance comparison of amorphous $Cs_3Bi_2I_9$ with some common amorphous (α) materials.

**Materials**	**Wavelength (nm)**	**Bias (V)**	**Responsivity (mA/W)**	**Response time**	**Ref**
α-Si	850	0.5	37	—	[11]
α-Si	320	2.3	118	—	[12]
α-Si/Ti	900	0	930	—	[13]
α-Si	525	220	280	—	[14]
α-Si	1300	0.8	0.053	—	[15]
α-Se	468	120	450	50/60 μs	[16]
α-Ge	1550	5	2	—	[17]
α-Ge	1550	4	5	—	[18]
α-Ge	1300	0.2	150	—	[19]
α-GeSn	633	2	0.041	—	[20]
α-MoS_2	1550	10	47.5	10/16 ms	[21]
α-$Cs_3Bi_2I_9$	1650	10	6.81	95/96 ms	This work

Table S4. The crystal structure of $Cs_3Bi_2I_9$ calculated by different methods.

Persudo potential	Cell parameters (Å)			Cell volume (Å^3)
	a	b	c	
PBE	8.63913	8.63913	22.15618	1432.0733
optB86b-vdW	8.38039	8.38039	21.11600	1284.3138
optB88-vdW	8.42672	8.42672	21.21844	1304.8524
vdW-DF2	8.08948	8.08948	20.07792	1137.8642
vdW-DF3	8.52032	8.52032	21.50915	1352.2786
Experiment	8.41	8.41	21.18	1297.32

References

[1] V. Petříček, M. Dušek, L. Palatinus, *Z. Krist.* **2014**, *229*, 345-352.

[2] W.-G. Li, X.-D. Wang, J.-F. Liao, Y. Jiang, D.-B. Kuang, *Adv. Funct. Mater.* **2020**, *30*, 1909701.

[3] Z. Li, X. Liu, C. Zuo, W. Yang, X. Fang, *Adv. Mater.* **2021**, *33*, 2103010.

[4] J. Wang, Y. Li, L. Ma, G. Shen, Q. Yang, *Adv. Funct. Mater.* **2022**, *32*, 2203072.

[5] Z. Qi, X. Fu, T. Yang, D. Li, P. Fan, H. Li, F. Jiang, L. Li, Z. Luo, X. Zhuang, A. Pan, *Nano Res.* **2019**, *12*, 1894-1899.

[6] X.-W. Tong, Z.-X. Zhang, D. Wang, L.-B. Luo, C. Xie, Y.-C. Wu, *J. Mater. Chem. C* **2019**, *7*, 863-870.

[7] K. M. Sim, A. Swarnkar, A. Nag, D. S. Chung, *Laser Photonics Rev.* **2018**, *12*, 1700209.

[8] D. Wu, H. Zhou, Z. Song, R. Liu, H. Wang, *J. Mater. Chem. C* **2018**, *6*, 8628-8637.

[9] A. Waleed, M. M. Tavakoli, L. Gu, S. Hussain, D. Zhang, S. Poddar, Z. Wang, R. Zhang, Z. Fan, *Nano Lett.* **2017**, *17*, 4951-4957.

[10] J. Zeng, H. Zhou, R. Liu, H. Wang, *Sci. China. Mater.* **2019**, *62*, 65-73.

[11] B. Desiatov, M. Lončar, *Appl. Phys. Lett.* **2019**, *115*, 121108.

[12] D. S. Schneider, A. Bablich, M. C. Lemme, *Nanoscale* **2017**, *9*, 8573-8579.

[13] F. Djeffal, N. Boubiche, H. Ferhati, J. Faerber, F. Le Normand, N. Javahiraly, T. Fix, *J. Alloy. Compd.* **2021**, *876*, 160176.

[14] S. Ghanbarzadeh, S. Abbaszadeh, K. S. Karim, *IEEE Electron Device Lett.* **2014**, *35*, 235-237.

[15] N. Kaplan, M. Grajower, N. Mazurski, J. Shappir, U. Levy, in *2017 Conference on Lasers and Electro-Optics (CLEO)*, **2017**, pp. 1-2.

[16] K. Wang, F. Chen, N. Allec, K. S. Karim, *IEEE Trans. Electron Devices* **2010**, *57*, 1953-1958.

[17] S. Mirbaha, R. N. Tait, in *Proc.SPIE, Vol. 7750*, **2010**.

[18] Y. Salamin, P. Ma, A. Emboras, Y. Fedoryshyn, B. Cheng, C. Hafner, J. Leuthold, in *Conference on Lasers and Electro-Optics*, Optica Publishing Group, San Jose, California, **2017**, p. STu1N.2.

[19] O. Jungwoo, S. K. Banerjee, J. C. Campbell, *IEEE Photonics Technol. Lett.* **2004**, *16*, 581-583.

[20] F. Yasar, W. Fan, Z. Ma, *IEEE Photonics J.* **2018**, *10*, 1-9.

[21] Z. Huang, T. Zhang, J. Liu, L. Zhang, Y. Jin, J. Wang, K. Jiang, S. Fan, Q. Li, *ACS Appl. Electron. Mater.* **2019**, *1*, 1314-1321.
